# Trace elements and tumors: homeostatic networks, molecular mechanisms and new horizons for precision intervention

**DOI:** 10.1186/s12943-026-02754-y

**Published:** 2026-07-30

**Authors:** Shengxin Zhang, Keqin Tan, Qin Zhang, Yuzhe Zhao, Ziyang Cheng, Xinggang Yang, Jingsi Dong, Jiaming Liu, Xuelei Ma

**Affiliations:** 1 Department of Biotherapy, Cancer Center and State Key Laboratory of Biotherapy, West China Hospital, Sichuan University, Chengdu, Sichuan China; 2 Department of Postgraduate Students, West China Hospital, Sichuan University, Chengdu, Sichuan China; 3 West China Hospital of Stomatology, Sichuan University, Chengdu, Sichuan China; 4 Department of Hematology, Children’s Hospital of Soochow University, Suzhou, Jiangsu China; 5 Department of Respiratory and Critical Care Medicine, Institute of Respiratory Health, Frontiers Science Center for Disease-Related Molecular Network, West China Hospital, Sichuan University, Chengdu, China; 6 Lung Cancer Center/Lung Cancer Institute, West China Hospital, Sichuan University, Chengdu, Sichuan China; 7 Department of Thoracic Surgery, West China Hospital, Sichuan University, Chengdu, Sichuan China; 8 Department of Urology, West China Hospital, Sichuan University, Chengdu, China

**Keywords:** Trace elements, Tumorigenesis, Ferroptosis, Cuproptosis, Tumor immune microenvironment, Metabolic reprogramming, Precision medicine, Element-responsive nanoplatforms, Synthetic biology

## Abstract

Trace elements including iron, copper, zinc, selenium, and manganese are indispensable for life, serving as enzyme cofactors, structural components, and signaling molecules that govern energy metabolism, redox balance, and genomic stability. Their physiological functions exhibit a characteristic dose–effect biphasicity, wherein homeostatic imbalance can both safeguard health and promote malignant transformation. This review systematically delineates the complete lifecycle of these five core trace elements under physiological conditions, emphasizing their extensive functional interconnections, and elucidates how tumor cells hijack this regulatory network through an element-addiction mechanism. We detail the emerging roles of iron-dependent ferroptosis and copper-dependent cuproptosis, alongside the context-dependent regulation of apoptosis and autophagy by trace elements, revealing tumor-specific metabolic vulnerabilities. Within the tumor immune microenvironment, we examine how zinc, iron, copper, selenium, and manganese function as molecular switches: zinc and selenium generally support antitumor immunity, whereas iron and copper dysregulation drives immunosuppressive polarization; manganese additionally emerges as a cGAS-STING agonist with immunotherapeutic potential. In tumor angiogenesis and metastasis, we explore the central roles of copper and iron in extracellular matrix remodeling, epithelial-mesenchymal transition, and angiogenic signaling. Extensive clinical evidence is integrated to establish systemic and local elemental imbalances as risk biomarkers across diverse cancer types. Therapeutic strategies are categorized into traditional nutritional and pharmacological interventions, element-metabolism-based synergistic sensitization with chemotherapy, radiotherapy, and immunotherapy, and interdisciplinary engineering approaches including element-responsive nanoplatforms and synthetic biology-engineered immune cells. Throughout, we emphasize that the majority of therapeutic evidence derives from preclinical models, underscoring that rigorous foundational research must precede clinical translation. We conclude by outlining knowledge gaps and priorities for future investigation, advocating a systems biology framework that acknowledges current limitations while guiding hypothesis-driven exploration.

**Supplementary Information:**

The online version contains supplementary material available at https://doi.org/10.1186/s12943-026-02754-y.

## Introduction

As one of the top global health threats, cancer continues to see rising incidence and mortality rates, imposing a heavy burden on human health, healthcare systems, and socioeconomic development worldwide. According to data from the World Health Organization (WHO), there were approximately 19.3 million new cancer cases and nearly 10 million cancer-related deaths globally in 2020. Driven by factors such as population aging, lifestyle transformations, and environmental influences, these figures are still on the rise, with new cases projected to reach 28.4 million by 2040 [1].

Different from the traditional understanding of local lesions, modern oncology research clearly defines cancer as a systemic, multifactorial, multi-step progressive metabolic disorder [2]. Its initiation and progression involve a complex regulatory network across multiple dimensions, including genomic mutations, metabolic reprogramming, and immune microenvironment dysregulation [2]. Tumor cells disrupt normal physiological balance to acquire malignant phenotypes such as unlimited proliferation, apoptosis resistance, invasion, metastasis, and immune evasion. Notably, significant heterogeneity exists across different cancer types, among individual patients, and even within distinct regions of the same tumor [3]. This has created a bottleneck for traditional single-target therapeutic strategies, highlighting an urgent need to explore new intervention directions from more systematic and fundamental physiological regulatory networks.

Trace elements refer to a class of elements that are indispensable for maintaining life activities despite a daily requirement of less than 100 mg in the human body, including iron, copper, zinc, selenium, manganese, cobalt, and others [4]. Despite their extremely low concentrations in the human body, these elements play irreplaceable core roles: serving as key cofactors of enzymes to participate in basic biochemical reactions such as energy metabolism, DNA replication and repair [5]; acting as structural support components to maintain the stable spatial conformation of proteins and nucleic acids [6]; and functioning as signaling molecules to regulate critical physiological processes including cell proliferation, differentiation and apoptosis [7]. Together, they constitute the core components of the "life network" that sustains cellular homeostasis and organismal health.

Importantly, the physiological functions of trace elements exhibit a typical dose–effect biphasicity, acting as a double-edged sword: within an optimal concentration range, they safeguard homeostasis, whereas deviations toward deficiency or excess can disrupt regulatory networks and promote malignant transformation. This hormetic property underscores the necessity of precisely maintaining trace element homeostasis. Not surprisingly, tumor cells have evolved to hijack this regulatory network, reshaping elemental metabolism to fuel their malignant proliferation [7–9].

This review adheres to the core logical thread of “homeostatic imbalance, then core mechanisms, and ultimately targeted intervention” to systematically integrate fragmented research findings and construct a comprehensive theoretical framework for the interactions between trace elements and cancer. Firstly, it deciphers the regulatory network governing the absorption, transport, storage, and excretion of trace elements under physiological conditions, as well as the mechanisms by which tumor cells hijack this network. Secondly, it elaborates on the core molecular mechanisms through which trace elements reshape the tumor immune microenvironment and drive metabolic reprogramming and genomic instability by regulating cell death fates such as ferroptosis, cuproptosis, and apoptosis. Furthermore, it summarizes the translational potential of trace elements in tumor risk prediction, therapeutic target screening, and combination therapy. Finally, it discusses the application prospects of multi-omics technologies, nanomedicine, and organoid models in this field.

## Trace element homeostasis regulation and tumor-driven homeostasis hijacking

Under physiological conditions, the homeostasis of trace elements is maintained through a sophisticated and multi-layered regulatory network that ensures essential elements such as iron, copper, zinc, and selenium are kept within narrow concentration windows [10]. Rather than presenting a fragmented, element-by-element account across separate functional categories, the following sections systematically describe the complete lifecycle, including absorption, transport, storage, and excretion for each major trace element [11]. We focus on the central trace elements iron (Fe), copper (Cu), zinc (Zn), selenium (Se), and manganese (Mn), which have the most established and mechanistically detailed roles in tumor biology. Where appropriate, we highlight functional interconnections among these elements, such as shared uptake pathways and enzyme cofactor dependencies.

### Iron (Fe)

#### Absorption

Intestinal iron absorption occurs primarily in the duodenum through two parallel pathways: heme iron enters enterocytes via mechanisms that remain unclear and is then decomposed into Fe^2^⁺ by heme oxygenase‑1 (HO-1); while non‑heme iron (predominantly Fe^3^⁺) is first reduced to Fe^2^⁺ by the brush‑border ferrireductase DcytB, and also by certain STEAP family metalloreductases, then taken up by divalent metal transporter 1 (DMT1) [12]. The absorbed Fe^2^⁺ is exported across the basolateral membrane by ferroportin and oxidized to Fe^3^⁺ by hephaestin (a ceruloplasmin homolog) before being loaded onto transferrin. Approximately 1–2 mg of iron is absorbed per day, representing a key regulatory step in systemic iron homeostasis [10, 13].

#### Transport

Once inside the enterocyte, iron can either be stored within ferritin or exported [14]. Export is mediated by ferroportin 1 (FPN1), the sole known mammalian iron exporter. After export, Fe^2^⁺ is re‑oxidized to Fe^3^⁺ by hephaestin (in enterocytes) or by the homologous copper‑dependent ceruloplasmin (in hepatocytes and macrophages), directly linking iron export to copper status [15]. Circulating Fe^3^⁺ is tightly bound to transferrin (Tf), which delivers iron to target cells via transferrin receptor 1 (TfR1/CD71)-mediated endocytosis [14, 16].

#### Storage

Normally, over 70% of body iron is in hemoglobin and myoglobin, and about 25% is stored as ferritin and hemosiderin in the liver, spleen, and bone marrow. Under severe iron overload, iron also deposits as hemosiderin, an insoluble and partially degraded ferritin, in lysosomes of macrophages, hepatocytes, and bone marrow, which are the main iron storage organs [17, 18].

#### Excretion

Unlike many other trace elements, humans do not have an active and regulated mechanism for iron excretion, so the iron content in the body is mainly controlled through absorption rather than clearance [19]. Iron loss occurs passively through daily shedding of intestinal epithelial cells, minimal blood loss (e.g., gastrointestinal bleeding or menstruation), and, to a lesser extent, biliary and urinary excretion [20]. Total daily iron loss in healthy adults is approximately 1–2 mg, which is matched by an equivalent amount of intestinal iron absorption to maintain systemic iron homeostasis [21].

### Copper

#### Absorption

The absorption of dietary copper mainly occurs in the duodenum and small intestine [22]. Copper is taken up by the high-affinity copper transporter CTR1 (SLC31A1), which is located on the apical membrane of enterocytes and is specific for Cu⁺. Therefore, Cu^2+^ in food must first be reduced to Cu^+^ by metalloreductases before being imported by CTR1 [12, 23]. This reduction is primarily carried out by STEAP family metalloreductases on the apical membrane of enterocytes [24, 25]. These same metalloreductases (STEAP2‑4) can also reduce ferric iron (Fe^3^⁺) for iron absorption.

#### Transport

Following uptake, copper (Cu^+^) is rapidly bound to intracellular copper chaperones to prevent free copper toxicity and to direct copper to specific subcellular destinations. The chaperone antioxidant protein 1 (ATOX1) delivers copper to the trans-Golgi network (TGN), where it is inserted into copper-dependent enzymes such as the ferroxidase ceruloplasmin and the copper-transporting ATPases (ATP7A and ATP7B) [26, 27]. When copper is excessive, ATOX1 promotes ATP7A/7B trafficking from TGN to peripheral vesicles for copper export: ATP7A exports copper to blood (intestine) or reabsorption pool (kidney), while ATP7B exports copper to bile (liver) or milk (mammary gland), maintaining copper homeostasis [28]. Once in the circulation, Cu⁺ is rapidly oxidized to Cu^2^⁺ by ceruloplasmin and other oxidases, allowing it to be stably bound by transport proteins [29]. In the circulation, Cu^2^⁺ is primarily bound to ceruloplasmin, while smaller exchangeable fractions associate with albumin, histidine, and α₂‑macroglobulin, delivering copper first to the liver/kidneys and then to peripheral tissues. At peripheral tissues, exchangeable Cu^2^⁺ is reduced to Cu⁺ by metalloreductases and imported via CTR1, while ceruloplasmin-bound Cu^2^⁺ acts as a reserve pool that sustains the exchangeable fraction through dynamic dissociation [30].

#### Storage

In the body, copper is mainly stored in the liver. In liver cells, copper can bind to metallothionein (MT) and exist in a storage form, preventing oxidative stress damage caused by free copper ions. In addition, copper chaperone protein CCS delivers copper to superoxide dismutase 1 (SOD1) to clear reactive oxygen species and maintain copper homeostasis; cytochrome c oxidase copper chaperone 17 (COX17) delivers copper to cytochrome c oxidase (COX) in mitochondria for oxidative phosphorylation and mitochondrial function. The liver serves as the primary reservoir for body copper, containing approximately 10–15% of total body copper in a healthy adult, with the remainder distributed in brain, kidney, heart, and other tissues [31].

#### Excretion

The clearance of copper is mainly carried out through hepatic and biliary secretion, accounting for about 80% of the daily excretion; in liver cells, ATP7B transports copper to bile and excretes it with feces. This pathway is regulated by copper status-excretion increases at high intake and decreases at low intake [32]. Urinary excretion plays a minor role (10–50 μg/day) under normal conditions but may increase substantially in copper overload states such as Wilson disease; losses through sweat, hair, and nails are quantitatively negligible for systemic copper homeostasis [32].

### Zinc

#### Absorption

Zinc absorption occurs throughout the small intestine (highest in the jejunum, lower in the duodenum and ileum), where zinc enters enterocytes via the apical importer ZIP4 (Zrt/Irt-like protein 4; encoded by SLC39A4). ZIP4 expression is feedback-regulated by intracellular zinc levels: upon deficiency, ZIP4 translocates to the apical membrane to enhance uptake, whereas under sufficiency, it is internalized and degraded [33].

#### Transport

Once inside the enterocyte, zinc enters the intracellular labile zinc pool and can follow one of several fates: binding to MT for buffered storage, transport to organelles, or export across the basolateral membrane [34]. Export across the basolateral membrane into the portal circulation is mediated by the zinc exporter ZnT1 (SLC30A1), which is the primary zinc efflux transporter in enterocytes and is upregulated by high intracellular zinc levels [35]. Zinc entering the bloodstream mainly binds to albumin, with a small amount binding to α₂-macroglobulin, transferrin, etc., and is transported in an exchangeable form to various tissues throughout the body, with the liver being the core metabolic organ for zinc.

At the systemic level, zinc transport is bidirectional and closely regulated by the coordinated action of 14 ZIP transporters (SLC39A family) and 10 ZnT transporters (SLC30A family): ZIP transporters mediate zinc input from the extracellular space or organelles to the cytoplasm, while ZnT transporters mediate zinc output from the cytoplasm to organelles or extracellular space [36]. This dual transporter system allows cells to precisely control cytoplasmic zinc concentrations over a remarkably wide range (from picomolar to millimolar levels) and to generate transient zinc signals that regulate diverse cellular processes including proliferation, differentiation, and apoptosis.

#### Storage

In a healthy adult, the total amount of zinc in the body is approximately 1.5–2.5 g. The vast majority (about 85–90%) of zinc is stored in skeletal muscles and bones, with skeletal muscles accounting for about 57% and bones accounting for about 29%. The remaining zinc is distributed in tissues such as the skin (about 6%) and liver (about 5%), with the prostate being one of the soft tissues with the highest zinc concentration [37]. Within cells, zinc is sequestered into intracellular vesicles via ZnT2, ZnT3, ZnT4, and ZnT8, which transport zinc into endosomes, synaptic vesicles, secretory granules, and insulin granules, respectively [38]. MTs serve as the primary intracellular zinc buffer, with each MT molecule capable of binding up to 7 zinc ions through its 20 cysteine thiolate groups, and they can rapidly release zinc under conditions of oxidative stress or upon specific signaling inputs [39].

#### Excretion

The human body lacks an active mechanism for regulating zinc excretion, and zinc loss mainly relies on passive pathways. Feces (excreted through bile) and urine are the two main pathways for zinc excretion, with sweat, menstruation, shed skin cells, and hair making relatively small contributions. The liver is a key organ for endogenous zinc excretion into bile. Liver cells actively pump cytoplasmic zinc into bile through ZnT1 (SLC30A1) located on the bile duct membrane, and then excrete it with feces [40]. In the kidneys, zinc is filtered by glomeruli and mostly reabsorbed in proximal tubules. ZIP10 on the brush border takes up zinc from urine into tubular cells, while ZnT1 on the basolateral membrane recycles zinc back into the bloodstream. High zinc intake upregulates ZnT1, promoting urinary zinc excretion [34]. There is a dynamic balance between zinc excretion and intake: during a high zinc diet, intestinal absorption decreases and fecal and urinary zinc excretion increases; during a low zinc diet, intestinal absorption increases, bile and urine zinc excretion decrease, and zinc storage in bones and liver is mobilized to maintain homeostasis.

### Selenium

#### Absorption

Selenium is absorbed with remarkable efficiency throughout the small intestine, with absorption rates exceeding 80% for most dietary forms, regardless of whether they are organic or inorganic [11].

Selenomethionine is absorbed via intestinal methionine transporters, enters the methionine pool, and is randomly incorporated into proteins in place of methionine at a low rate. Selenocystine is taken up by dibasic and neutral amino acid transporters, though its post-absorption fate is poorly understood. Selenite absorption is diet-dependent and varies from moderate to high, whereas selenate absorption is nearly complete; however, selenate must be reduced to selenite for further metabolism, and some is lost directly in urine [11]. Additionally, selenium lacks a dedicated basolateral efflux transporter in enterocytes. Selenomethionine is released via amino acid transporters, whereas selenite-derived selenide (HSe⁻) exits mainly by passive diffusion and is rapidly cleared by the liver upon entering portal blood [41].

#### Transport

In blood, selenium is transported in multiple forms with distinct kinetics and tissue targeting. Selenomethionine derived from dietary sources resembles methionine and can be nonspecifically incorporated into general body proteins (e.g., albumin) in place of methionine, forming a large but metabolically passive selenium reservoir that is not subject to homeostatic regulation. The main high‑affinity selenium transporter is Selenoprotein P (SELENOP), a unique glycoprotein containing multiple selenocysteine residues, which is primarily synthesized in the liver [42].

#### Storage

Unlike iron (ferritin) and zinc/copper (MT), selenium has no dedicated storage protein. Instead, the functional selenium reservoirs are the selenoproteins themselves, a diverse family encompassing glutathione peroxidase (GPX) 1‑4, thioredoxin reductases (TXNRD) 1‑3, deiodinases, and SELENOP, with at least 25 selenoproteins identified in humans [43]. Among these, SELENOP serves as the major plasma transport and storage form, while stress‑related selenoproteins such as GPX1 and SELENOW represent tissue‑specific functional pools that are dynamically regulated by selenium availability, rather than serving as storage depots [44, 45]. It is important to note that while dietary selenomethionine can be nonspecifically incorporated into general proteins in place of methionine, this occurs only when selenium is ingested in the form of selenomethionine, as humans lack the capacity to synthesize selenomethionine from inorganic selenium or selenocysteine [11, 46].

#### Excretion

Excess selenium is primarily excreted in urine after hepatic methylation via the pathway from selenide to methylselenol to dimethylselenide (a small amount exhaled) and finally to trimethylselenonium and selenosugars (excreted in urine), with urinary excretion as the major route while feces (from unabsorbed selenium and biliary secretion) and exhaled air play minor roles, and enterohepatic circulation exists but its flux is relatively small [47, 48].

### Manganese

#### Absorption

The absorption of manganese occurs throughout the small intestine, with the highest efficiency in the duodenum and proximal jejunum, but the overall absorption efficiency is relatively low, typically 3–5% of dietary intake under normal conditions. DMT1 (SLC11A2) is the primary apical transporter for Mn^2^⁺, although it also serves as the main transporter for ferrous iron (Fe^2^⁺), establishing a competitive interaction between manganese and iron absorption [49]. The transferrin/transferrin receptor pathway and voltage-gated calcium channels also contribute to cellular manganese uptake, particularly in non-intestinal tissues, highlighting the pleiotropic nature of manganese transport [50]. Therefore, manganese absorption is homeostatically regulated: it decreases when stores are sufficient and increases during deficiency, though the molecular details are less understood than for iron. The basolateral exit of manganese from enterocytes lacks an identified specific exporter and likely involves shared divalent metal transporters or unknown pathways, remaining a major gap in manganese homeostasis.

#### Transport

In the blood, manganese binds to several carrier proteins, mainly transferrin (which binds Mn^3^⁺ after its oxidation from Mn^2^⁺ by ceruloplasmin), α₂-macroglobulin, and albumin. The distribution of these carriers varies with manganese concentration and physiological status [51]. Cellular uptake of manganese from transferrin occurs via TfR1-mediated endocytosis, similar to iron uptake, providing a mechanism for manganese delivery to dividing cells that highly express TfR1 [52].

#### Storage

The manganese content in the adult body is about 12–20 mg, distributed in various tissues and organs. The parts with the highest manganese concentration in the human body are the liver, kidneys, and pancreas [53]. Within cells, manganese is predominantly localized in mitochondria, where it serves as an essential cofactor for manganese superoxide dismutase (MnSOD, encoded by SOD2), the primary antioxidant enzyme in the mitochondrial matrix that converts superoxide radicals to hydrogen peroxide [54].

#### Excretion

**Fig. 1:**
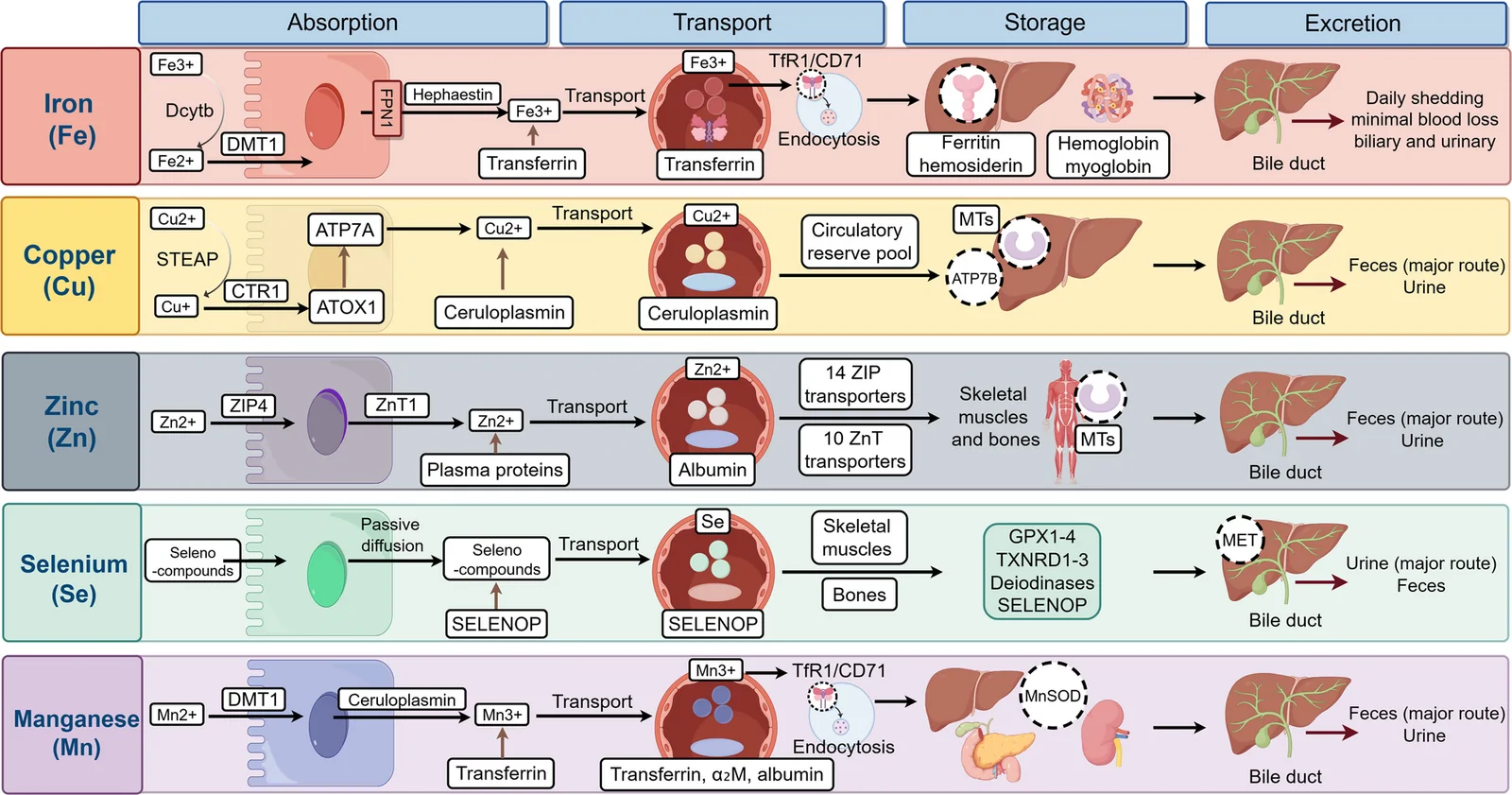
Physiological regulatory networks of essential trace element homeostasis. Note. This figure depicts the complete lifecycle of Fe, Cu, Zn, Se, and Mn across absorption, transport, storage, and excretion. Fe and Mn share DMT1 uptake and Tf/TfR1-mediated delivery, with Fe stored as ferritin and Mn in mitochondria (MnSOD cofactor); Cu enters via CTR1 and is cleared chiefly by biliary ATP7B, while Zn uses ZIP/ZnT transporters, with most stored in muscle/bone. Se is absorbed with high efficiency as various forms, transported by SELENOP, incorporated into selenoproteins, and excess methylated for urinary excretion. For all five elements, the liver serves as the metabolic hub, and homeostasis is regulated primarily at absorption and biliary excretion; solid arrows denote major fluxes, dashed lines minor or passive routes. Abbreviations: ATOX1, antioxidant 1 copper chaperone; ATP7A/ATP7B, copper-transporting ATPases; α₂M, α₂-macroglobulin; CCS, copper chaperone for superoxide dismutase; COX17, cytochrome c oxidase copper chaperone; CTR1, copper transporter 1; Cu, copper; DcytB, duodenal cytochrome B; DMT1, divalent metal transporter 1; Fe, iron; FPN1, ferroportin 1; GPX1–4, glutathione peroxidase 1–4; HO‑1, heme oxygenase‑1; Mn, manganese; MnSOD, manganese superoxide dismutase; MT, metallothionein; Se, selenium; SeCys₂, selenocystine; SELENOP, selenoprotein P; SeMet, selenomethionine; SOD1, superoxide dismutase 1; SOD2, superoxide dismutase 2; STEAP, six-transmembrane epithelial antigen of the prostate; Tf, transferrin; TfR1, transferrin receptor 1; TXNRD1–3, thioredoxin reductase 1–3; ZIP, Zrt/Irt-like protein; Zn, zinc; ZnT, zinc transporter

Manganese excretion occurs primarily via the biliary route, with the liver secreting manganese into the bile, from which it is eliminated in the feces, accounting for approximately 90% of total manganese excretion. Biliary manganese is secreted in both free ionic form and bound to glutathione and other small-molecule complexes, and this process is saturable, such that high manganese loads can exceed biliary excretory capacity, leading to tissue accumulation and potential toxicity [51]. Renal excretion of manganese accounts for a minor fraction (approximately 10% or less) of total elimination, with manganese filtered by the glomerulus and partially reabsorbed by the renal tubules, resulting in low urinary manganese levels under normal conditions. The complete physiological lifecycles of these five essential trace elements, from intestinal absorption through systemic transport and tissue storage to biliary or urinary excretion, are summarized in Fig. 1.

## Trace element-driven cell death

### Ferroptosis: Iron‑dependent metabolic vulnerability

Ferroptosis is an iron-dependent and regulated form of cell death, discovered in 2012 and named for its iron-dependent characteristics. Its core biochemical hallmark is the lethal accumulation of iron-catalyzed lipid peroxides on the cell membrane, resulting in morphological and biochemical characteristics distinct from apoptosis, necrosis, and autophagy [55]. The core mechanism of this process lies in the collapse of the cellular antioxidant defense system (especially the glutathione-dependent lipid repair system). This creates a key metabolic weakness for many cancer cells, which usually exhibit abnormal iron metabolism and increased oxidative stress [56].

Mechanistically, intracellular iron catalyzes the Fenton reaction: Fe^2^⁺ reacts with H₂O₂ to generate hydroxyl radicals, which initiate lipid peroxidation [57]. A key enzyme in this process is ACSL4 (acyl-coenzyme A synthetase long-chain family member 4), which esterifies long-chain fatty acids into membrane phospholipids, increasing the pool of polyunsaturated fatty acids that are susceptible to peroxidation. Specifically, polyunsaturated fatty acids (PUFAs) are first activated by ACSL4 to form long-chain acyl-CoA [58, 59]. Lysophosphatidylcholine acyltransferase 3 (LPCAT3) then transfers the acyl group to membrane phosphatidylethanolamine (PE), generating PUFA-PE, the substrate for lipoxygenase-mediated lipid peroxidation [60]. High ACSL4 expression sensitizes cancer cells to ferroptosis, positioning it as a key regulator and a potential therapeutic target [61].

Ferroptotic cells exhibit characteristic mitochondrial contraction, increased membrane density, and decreased matrix [62], accompanied by a significant elevation of lipid reactive oxygen species (ROS). These features, together with the iron-dependent mechanism described above, distinguish ferroptosis from apoptosis, necrosis, and autophagy [63]. Consequently, cancer cells with dysregulated iron metabolism, characterized by increased iron uptake and storage, exhibit heightened susceptibility to ferroptosis, a vulnerability that can be therapeutically exploited [64, 65].

The central defense against ferroptosis is the system Xc⁻/glutathione (GSH)/GPX4 axis. System Xc⁻ imports cystine for GSH synthesis; GSH serves as the essential cofactor for GPX4, which reduces lipid peroxides and prevents their accumulation [56]. When system Xc⁻ is inhibited, intracellular GSH levels decrease, GPX4 activity is reduced, and lipid peroxides accumulate, thereby promoting the occurrence of ferroptosis [66]. For example, the chemical erastin reduces intracellular cysteine by inhibiting system Xc⁻ and depletes GSH, which in turn triggers ferroptosis through the dysfunction of GPX4 [67].

Therefore, the iron-dependent metabolic vulnerability of ferroptosis offers a promising therapeutic avenue, particularly for tumors that have acquired resistance to conventional therapies [68].

### Cuproptosis: A Newly Defined Form of Copper‑Dependent Cell Death

Cuproptosis is a new programmed cell death mode established in recent years, whose mechanism is fundamentally different from the known pathways [69]. The core mechanism of cuproptosis is that excessive copper ions (mainly Cu⁺) do not cause extensive oxidative damage but precisely target lipoylated proteins in the mitochondrial tricarboxylic acid (TCA) cycle. This process is mediated by ferredoxin 1 (FDX1), which both catalyzes protein lipoylation and reduces Cu^2^⁺ to the more reactive Cu⁺ [69]. The reduced Cu⁺ specifically binds to lipoylated proteins, most notably dihydrolipoamide S‑acetyltransferase (DLAT), inducing their aberrant oligomerization and aggregation [70]. This proteotoxic stress destabilizes iron‑sulfur cluster proteins in mitochondria, disrupts the metabolic hub of the cell, and ultimately leads to energy collapse and cell death [70].

The execution of cuproptosis is profoundly dependent on the metabolic state of cells, showing distinct metabolic selectivity. Cells that are highly dependent on mitochondrial oxidative phosphorylation and the TCA cycle are extremely sensitive to the cuproptosis pathway [71]. However, cells that mainly rely on glycolysis for energy supply show significant tolerance [69, 71]. The molecular basis for this differential sensitivity lies in FDX1‑mediated protein lipoylation, which is a prerequisite for cuproptosis, and disruption of the lipoylation pathway confers complete resistance [69].

**Fig. 2:**
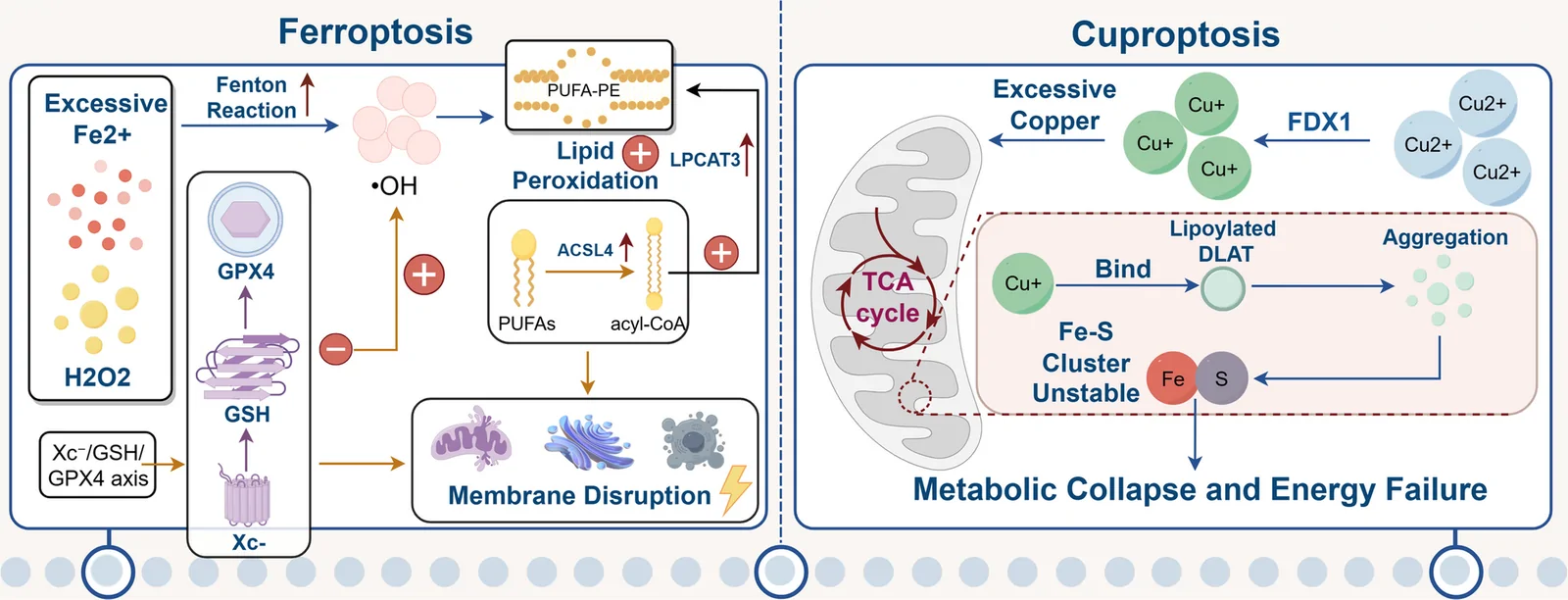
Sequential cascades of ferroptosis and cuproptosis. Note. This figure illustrates the cascades of two metal‑dependent cell death pathways organized into three chronological phases, with ferroptosis depicted in the left panel and cuproptosis in the right panel. For ferroptosis, the initiation phase shows the collapse of the system Xc⁻/GSH/GPX4 antioxidant axis; the execution phase highlights the Fenton reaction, in which ferrous iron reacts with hydrogen peroxide to generate hydroxyl radicals (·OH) that drive massive lipid peroxidation on polyunsaturated fatty acid‑containing phospholipids; and the degradation phase depicts mitochondrial shrinkage, ER dilatation, and plasma membrane rupture. For cuproptosis, the initiation phase shows FDX1‑mediated reduction of Cu^2^⁺ to Cu⁺ and protein lipoylation; the execution phase highlights Cu⁺ binding to lipoylated DLAT, inducing its aberrant aggregation and proteotoxic stress; and the degradation phase depicts TCA cycle dysfunction, ATP depletion, and cell death. All arrows point unidirectionally from the core execution events toward the terminal structural and metabolic consequences, confirming that organellar and energetic failure are downstream effects rather than initiating events in both pathways. Abbreviations: Fe, iron; Cu, copper; ·OH, hydroxyl radical; GPX4, Glutathione Peroxidase 4; ACSL4, Acyl-CoA Synthetase Long Chain Family Member 4; PUFA-PE, polyunsaturated fatty acid-containing phosphatidylethanolamine; LPCAT3, lysophosphatidylcholine acyltransferase 3; FDX1, Ferredoxin 1; TCA, Tricarboxylic Acid; mTOR, mechanistic Target of Rapamycin

In tumor biology, the discovery of cuproptosis has opened new therapeutic avenues. Although many cancer cells undergo metabolic reprogramming toward glycolysis (the Warburg effect), they often retain sufficient mitochondrial respiratory capacity to support anabolic metabolism, rendering them potentially susceptible to cuproptosis. Moreover, the tumor microenvironment (TME) is frequently characterized by copper dysregulation and accumulation, further sensitizing tumor cells to copper-dependent death. Thus, cuproptosis provides a new metabolic target for selective tumor killing [72]. Current strategies focus on enhancing copper accumulation in tumor cells using copper ionophores such as elesclomol and disulfiram‑copper complexes, or on directly activating the cuproptosis pathway by targeting key nodes such as FDX1 [69, 73]. The key pathways of ferroptosis and cuproptosis are summarized in Fig. 2.

### Precise Regulation of Apoptosis and Autophagy by Trace Elements

Trace elements play an irreplaceable role in maintaining cellular homeostasis. Subtle changes in their concentration and distribution can directly regulate core fate decisions such as cell survival, autophagy or apoptosis [74]. Importantly, this regulation is highly context-dependent. The same element can exert opposing effects, promoting or suppressing apoptosis/autophagy, depending on its concentration and the cellular state. Zinc is a core regulator of the execution phase of apoptosis. Depletion of zinc promotes apoptosis by relieving caspase-3 inhibition, whereas excessive zinc accumulation triggers apoptosis through the mitochondrial pathway [75, 76]. Additionally, zinc homeostasis is a critical regulator of autophagy. Deficiency in zinc induces protective autophagy primarily by activating AMPK, which in turn inhibits mTOR signaling [77].

In contrast, the regulatory network of copper is more complex. Excessive copper ions mainly produce ROS through the Fenton reaction, triggering oxidative stress and activating the JNK/p38 MAPK-caspase-3 cascade, thereby inducing apoptosis [78]. However, copper has been shown to inhibit autophagy under certain conditions by interfering with the activity of key autophagy proteins such as ATG4B and impairing LC3 processing and autophagosome‑lysosome fusion, highlighting a context‑dependent negative regulation of the autophagic process [79].

Iron homeostasis imbalance drives multiple cell death modes. Excessive iron accumulation triggers oxidative stress and caspase-3-dependent apoptosis, while also contributing to ferroptosis and pyroptosis via ROS production and gasdermin pore formation [80]. Conversely, iron deficiency may induce mitophagy as an adaptive response through hypoxia-inducible factor 1α (HIF-1α) stabilization [81]. Moreover, selenium mainly regulates the intracellular redox balance by acting as a component of important selenoproteins, such as GPX, thereby indirectly affecting apoptosis and autophagy [82, 83]. Together, these observations show that trace element regulation of apoptosis and autophagy is context‑dependent, arising from dynamic integration of concentration, redox, and cell‑type cues, which tumors exploit for survival.

## Trace elements in the tumor immune microenvironment

The dynamic balance of tumor immune microenvironment is finely regulated by local biochemical signals. Recent studies have revealed that essential trace elements such as zinc, iron, selenium, copper, and manganese play a key ‘element switch’ role. They serve not only as necessary cofactors for immune-related enzymes and proteins but also, through subtle changes in their concentration and distribution, as direct regulators of the activation, differentiation, and function of immune cells like molecular switches, thus profoundly affecting the process of anti-tumor immune response [84].

### Zinc signaling network

The regulation of the immune system by zinc commences at the inaugural stage of antigen presentation. Dendritic cells (DCs), the most potent professional antigen-presenting cells, rely heavily on zinc signals for their maturation, migration, and capacity to activate T cells [85]. Moreover, zinc supplementation enhances DC maturation, T‑cell activation, and anti‑tumor immunity via upregulating costimulatory molecules/major histocompatibility complex class II (MHC II) and interleukin-12 (IL-12) secretion, and is also essential for T‑cell development and function [86–88]. Beyond its immune effects, zinc also supports cellular metabolism and genomic stability by stabilizing transcription factors via zinc-finger motifs and serving as the catalytic center of superoxide dismutase (SOD) to combat oxidative stress [89, 90].

Zinc deficiency is a common feature of various cancers, which creates a favorable environment for tumor development by weakening immune surveillance, exacerbating oxidative damage, and disrupting transcriptional regulation, and is closely associated with an increased tumor risk [91–94]. For example, serum zinc levels are significantly reduced in patients with breast cancer, bladder cancer, and esophageal squamous cell carcinoma (ESCC) [91, 92, 95, 96]. This systemic deficiency reflects active zinc sequestration by tumor cells through upregulation of specific importers such as ZIP4, which depletes the circulating zinc pool [97]. Conversely, dysregulated zinc accumulation or transporter reprogramming can also promote cancer. For instance, ZIP7-mediated zinc influx in estrogen receptor (ER)‑positive breast cancer activates kinase signaling and drives progression, while ZnT2 upregulation sequesters zinc into vesicles, contributing to drug resistance [98, 99]. In esophageal squamous cell carcinoma, ZIP6‑mediated zinc influx promotes proliferation and metastasis [100]. Thus, zinc exhibits a critical duality: it maintains cellular order under homeostasis, yet its disruption through deficiency or dysregulation can promote tumor progression via multiple pathways.

### Iron metabolism

Iron metabolism plays a regulatory role in the tumor immune microenvironment. Its homeostatic imbalance is not only the driving factor of tumor cell proliferation, but also the key metabolic basis for immune cell function to be systematically remodeled [101]. At the tumorigenic level, iron at physiological levels acts as a cofactor for ribonucleotide reductase, ensuring DNA replication fidelity, and for catalase, participating in ROS clearance [102, 103]. However, cancer cells systematically sequester iron by overexpressing TfR1 and suppressing FPN1, expanding the labile iron pool [104]. This “iron addiction” fuels proliferation but also catalyzes hydroxyl radical production via Fenton chemistry, driving oxidative DNA damage and genomic instability [80]. Critically, this iron sequestration is not limited to tumor cells; it also depletes the interstitial bioavailable iron pool, creating an iron‑deficient microenvironment that redirects tumor‑associated macrophages (TAMs) toward an immunosuppressive M2‑polarized state. Specifically, M2 TAMs highly express CD163 and HO‑1 to acquire heme‑derived iron, store it in ferritin, and actively export it via FPN1, resulting in an iron‑deficient phenotype [104]. This iron deprivation and M2 polarization are driven by tumor cells through TfR‑mediated iron competition, as shown in hepatocellular carcinoma [105]. Consequently, the iron‑deprived milieu acts as an extrinsic cue that promotes M2 polarization. Reversing this via exogenous iron supplementation (such as using iron oxide nanoparticles) can repolarize TAMs toward a pro-inflammatory M1 phenotype with anti-tumor potential [106, 107].

Neutrophils are another class of key innate immune cells regulated by iron metabolism in the TME. They are not only an important source of Lipocalin-2, the secreted form of which can bind iron and affect the iron uptake and growth of tumor cells, in metastatic lesions, tumor cell-derived granulocyte macrophage colony-stimulating factor (GM-CSF) can also induce neutrophils to express and secrete transferrin, which directly stimulates the growth of metastatic tumor cells and forms a paracrine loop to promote metastasis [108, 109]. Iron metabolism also profoundly affects the function of neutrophils and their sensitivity to ferroptosis. On the other hand, neutrophils themselves may also undergo ferroptosis and release oxidized lipid molecules, thereby inhibiting the function of effector T cells and promoting immunosuppression [110, 111]. However, in some breast cancer models, neutrophils produce itaconic acid through high expression of aconitate decarboxylase 1 (ACOD1), which activates the antioxidant transcription factor nuclear factor erythroid 2-related factor 2 (Nrf2), thereby acquiring resistance to ferroptosis and maintaining their immunosuppressive function [112]. This difference highlights the complexity and context-dependence of iron metabolism in regulating neutrophil function.

Therefore, the imbalance of iron metabolism in the TME, especially the iron recovery, efflux and secretory programs with TAMs and neutrophils, constitutes a key “metabolic immune checkpoint”. By dynamically controlling the spatial distribution and utilization of iron, an essential resource, it precisely regulates the function and fate of innate immune cells, providing a scientific basis for immunotherapy strategies targeting iron metabolism.

### Copper metabolism

Copper is an essential trace element that serves as a cofactor for key enzymes (e.g., superoxide dismutase and cytochrome c oxidase), supporting energy metabolism, antioxidant defense, and signal transduction [113]. In the immune system, copper promotes T‑cell activation and function via T-cell receptor (TCR) signaling and the MAPK/PI3K‑Akt pathways; deficiency impairs T‑cell proliferation and responses [114, 115]. Beyond its physiological roles, the copper transporter ATP7A supplies cofactors to activate lysyl oxidase (LOX) family enzymes, driving tumor fibrosis, invasion, and metastasis, while also maintaining copper homeostasis in immune cells to sustain anti‑tumor immunity [116, 117]. As signaling molecules, copper ions directly upregulate programmed death-ligand 1 (PD-L1) expression; the uptake protein CTR1 positively correlates with PD‑L1 levels, and copper chelators reduce PD‑L1 while enhancing CD8⁺ T‑cell infiltration [118]. Copper also activates transforming growth factor beta activated kinase 1 (TAK1) to trigger nuclear factor-κB (NF-κB) signaling, which promotes PD‑L1 expression and transcriptionally represses CTR1 to maintain homeostasis; disruption of this feedback loop leads to dysregulated copper uptake and activates pro‑survival pathways, potentially mediating resistance to NF‑κB‑targeted therapy [119]. However, copper excess exerts potent protumor effects: it directly activates MEK1 to amplify MAPK signaling, stabilizes HIF to induce vascular endothelial growth factor (VEGF)‑driven angiogenesis, and catalyzes ROS production via Fenton‑like reactions, thereby exacerbating oxidative damage and driving malignant transformation [120–124]. Thus, copper homeostasis is a critical determinant of cellular fate. Physiological levels preserve cellular order, whereas excess subverts normal physiology through signaling and transcriptional dysregulation, contributing to a wide range of diseases including cancer.

### Selenium metabolism

Selenium is an essential trace element that exerts its biological functions primarily through selenoproteins, which mediate antioxidant defense (such as GPX/GPX4 and TXNRD), immune regulation, apoptosis induction, and inhibition of tumor progression [125, 126]. At physiological concentrations, selenium supports GPX4 expression to protect against ferroptosis [127]. Notably, pharmacological doses of selenium have been shown to further upregulate GPX4 through transcriptional activation via TFAP2c/Sp1, without inducing ferroptosis or ROS toxicity, suggesting a broad therapeutic window [127].

In terms of immunity, selenium enhances the activity and function of T cells and natural killer (NK) cells by maintaining intracellular redox balance via selenoprotein‑dependent ROS elimination, thereby protecting immune cells from oxidative damage [128–130]. Its anticancer effects also include inducing tumor cell apoptosis, blocking cell cycle progression, inhibiting matrix metalloproteinase-2 (MMP-2)/matrix metalloproteinase-9 (MMP-9) and urokinase-type plasminogen activator (uPA) to suppress invasion and metastasis, and upregulating selenoprotein expression to accelerate DNA repair and reduce mutation accumulation [131]. Epidemiologic studies have consistently shown an inverse association between selenium status and the risk of several cancers, most notably esophageal, gastric, colorectal, lung, and prostate cancer [132–134]. However, the role of selenium follows a U‑shaped dose‑response curve: deficiency weakens antioxidant defenses and increases cancer risk, whereas excessive supplementation can exert pro‑oxidant effects, disrupt calcium homeostasis, induce lipid peroxidation and caspase‑mediated apoptosis, and may even increase cancer mortality in certain contexts (e.g., high‑dose use after diagnosis) [132–136]. The net efficacy of selenium is highly dependent on its chemical form, dosage, baseline selenium status, genetic background, and cancer type, which collectively explain the heterogeneity across different study conclusions [137–140].

### Manganese metabolism

**Fig. 3:**
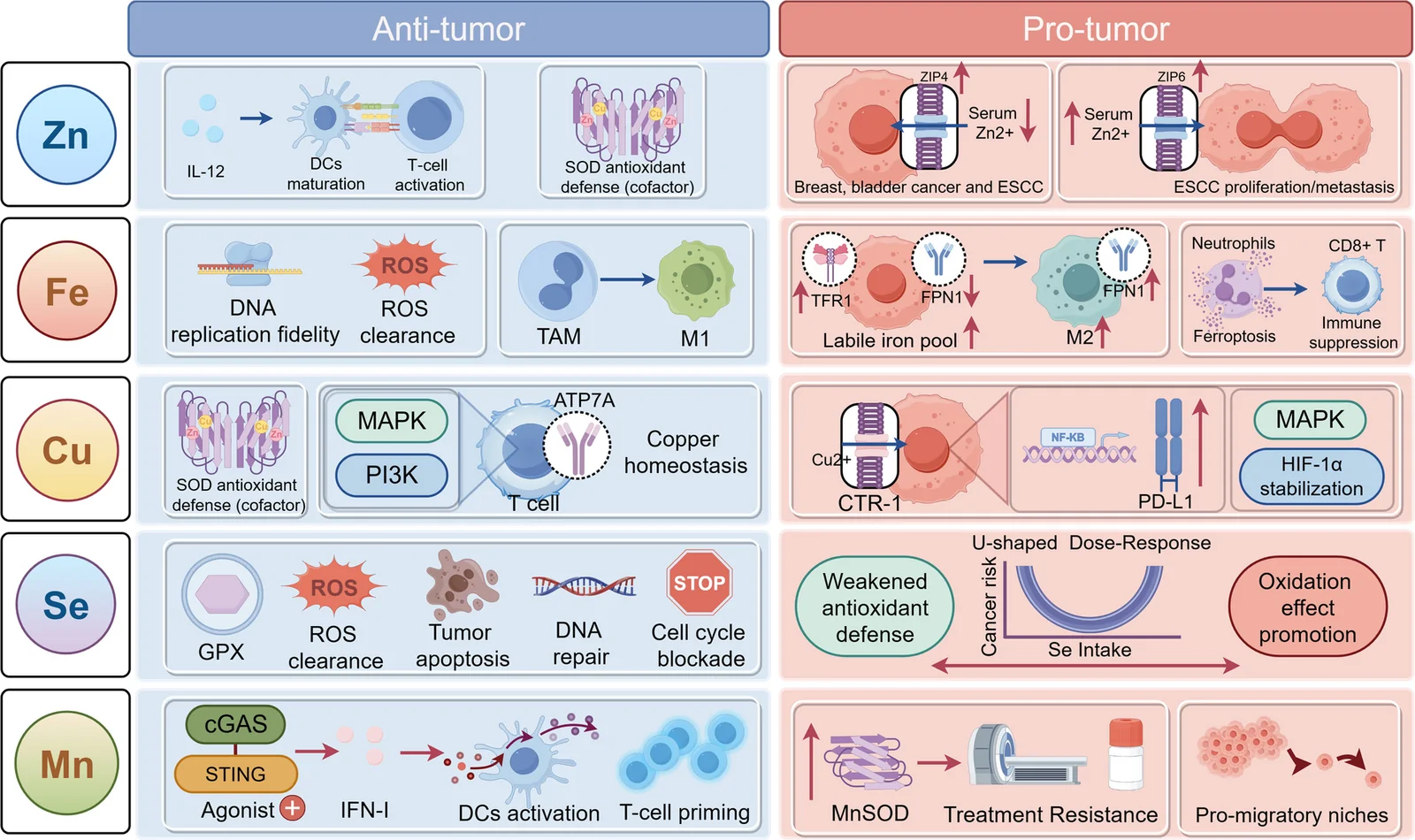
Dual roles of trace elements in the tumor immune microenvironment. Note. This figure illustrates the opposing roles of five essential trace elements, including Zinc (Zn), Iron (Fe), Copper (Cu), Selenium (Se), and Manganese (Mn) in modulating tumor progression and anti-tumor immunity. For Zn, deficiency increases cancer risk via ZIP4-mediated sequestration, while ZIP6-driven uptake promotes ESCC metastasis; conversely, Zn supports DC maturation and T-cell activation. For Fe, tumor cells overexpress TFR1 and suppress FPN1 to fuel proliferation and ROS-driven DNA damage, while depleting the microenvironment to polarize TAMs toward an immunosuppressive M2 phenotype. For Cu, it promotes immune evasion via NF-κB/PD-L1 upregulation, MAPK signaling, and HIF-1α stabilization, yet maintains immune cell homeostasis. For Se, its effects follow a U-shaped dose–response curve, with optimal levels supporting antioxidant defense (GPX) and DNA repair, while deficiency or excess promotes tumor growth. For Mn, it acts as a cGAS-STING agonist to boost anti-tumor immunity, yet as a MnSOD cofactor, it drives treatment resistance and pro-migratory niches. Abbreviations: Cu, copper; DCs, dendritic cells; ESCC, esophageal squamous cell carcinoma; Fe, iron; FPN1, ferroportin 1; GPX, glutathione peroxidase; HIF-1α, hypoxia-inducible factor 1-alpha; Mn, manganese; MnSOD, manganese superoxide dismutase; NF-κB, nuclear factor kappa B; PD-L1, programmed death-ligand 1; ROS, reactive oxygen species; Se, selenium; TAMs, tumor-associated macrophages; TFR1, transferrin receptor 1; Zn, zinc

Manganese is a cofactor for several essential enzymes, most notably MnSOD, the primary antioxidant enzyme in the mitochondrial matrix. MnSOD is upregulated in aggressive tumors such as glioblastoma, breast cancer, and colorectal cancer, contributing to resistance against radiotherapy and chemotherapy [141, 142]. Moreover, tumor growth systemically redistributes manganese to form pro-migratory niches, and cancer cells secrete Mn^2^⁺ via extracellular vesicles to enhance invasion. This effect can be blocked by low-dose heparin [143]. Moreover, environmental manganese exposure, particularly through drinking water contamination, has been associated with increased overall cancer risk, highlighting the double-edged nature of this element [144]. Recent discoveries have also unveiled a novel role for manganese in antitumor immunity. Manganese ions (Mn^2^⁺) act as potent agonists of the cGAS-STING pathway, amplifying type I interferon responses and promoting dendritic cell activation and T-cell priming. Preclinical studies show that manganese administration synergizes with immune checkpoint inhibitors (ICIs), leading to enhanced tumor rejection and long-term immune memory [145]. Clinical trials are underway to evaluate Mn^2^⁺ in combination with anti-programmed death 1 (PD-1)/PD-L1 therapy. Figure 3 highlights the dual nature of these trace elements, showing how each can both support and subvert antitumor immunity depending on the context, and detailed clinical study characteristics for each cancer type and trace element are summarized in Supplementary Table 1.

## Trace elements and tumor progression and metastasis

### Tumor Angiogenesis

Copper plays a central role in driving tumor angiogenesis. For instance, copper ions can directly bind to and stabilize HIF-1α, while enhancing the activity of NF-κB, thereby ensuring the transcriptional activation of key transcription factors such as VEGF and fibroblast growth factor 2 (FGF2), which are pro-angiogenic mediators [146–148]. Additionally, copper can directly interact with angiogenin (ANG), modulating its affinity for endothelial cells and thereby promoting angiogenesis [149, 150]. Furthermore, the pro-angiogenic effect of copper also relies on its homeostatic regulatory protein network, in which CTR1 not only mediates copper uptake but also forms a complex with vascular endothelial growth factor receptor 2 (VEGFR2) via disulfide bonds, driving its internalization and sustained downstream signaling activation, a critical step in angiogenesis [151]. Moreover, iron promotes tumor angiogenesis through various mechanisms. Firstly, HIFs are key regulatory factors in angiogenesis, and the availability of iron directly affects the stability and activity of HIFs [152]. In the hypoxic TME, HIF-1α accumulates and activates, upregulating the expression of angiogenic genes such as VEGF [153]. Iron ions (Fe^2^⁺) serve as essential cofactors for prolyl hydroxylases (PHDs), which mark hypoxia-inducible factors (HIFs) for degradation under normoxic conditions. Dysregulated iron metabolism in tumor cells can impair PHD activity, leading to HIF stabilization and enhanced angiogenic signaling. For example, in lung adenocarcinoma, loss of miR-145-5p upregulates ceruloplasmin (CP), which oxidizes Fe^2^⁺ and reduces intracellular Fe^2^⁺ availability, thereby inhibiting Fe^2^⁺-dependent PHDs activity. This results in abnormal stabilization of hypoxia-inducible factor 2α (HIF-2α) even under normoxia, subsequently activating VEGF-A expression and driving tumor angiogenesis. The “Fe^2^⁺-PHD-HIF-2α” regulatory axis highlights how disrupted iron metabolism promotes tumor progression by interfering with oxygen-sensing pathways [154]. Therefore, iron ions drive tumor angiogenesis through a dual mechanism: at the signaling level, they act as cofactors of PHDs to regulate the stability of HIFs, thereby controlling the transcription of pro angiogenic factors such as VEGF; at the level of nutritional metabolism, as an essential cofactor for key enzymes such as DNA synthesis, it directly supports the proliferation of endothelial cells and tumor cells [155].

In the TME, these two pathways are tightly coupled through the central role of TfR1, forming a self-amplifying positive feedback loop. Tumor cells and endothelial cells competitively upregulate TfR1 to seize limited iron resources in order to meet their proliferative demands. This "iron plundering" compels endothelial cells to increase their own TfR1 expression to enhance their proliferation and angiogenesis abilities, and adapt to the needs of tumor growth [156, 157].

### Tumor progression and metastasis

Besides angiogenesis, trace elements, particularly copper and iron, play critical roles in tumor invasion, matrix remodeling, and distant dissemination, among which matrix remodeling is an essential prerequisite. Copper orchestrates the remodeling of the tumor extracellular matrix (ECM), a process primarily mediated by the copper-activated enzyme LOX, which paves the way for tumor cell dissemination [158, 159]. The copper-dependent enzymes LOX/lysyl oxidase-like (LOXL) promote tumor migration by remodeling the physical microenvironment. They achieve this by stiffening the ECM through catalyzing cross-linking of its collagen and elastin fibers, making it more conducive to invasion [160–162]. Copper and LOX constitute a pro-metastatic positive feedback loop. LOX expression is induced by copper‑mediated HIF‑1α activation, while LOX family members can in turn promote HIF‑1α stabilization, thereby reinforcing tumor progression [121, 122, 124]. Beyond ECM remodeling, copper also drives the epithelial-mesenchymal transition (EMT) and facilitates the establishment of the premetastatic niche. For example, copper can directly activate the HIF-1α-Snail/Twist signaling axis, thereby initiating the EMT program and endowing tumor cells with the ability to migrate and invade [163]. Copper can directly promote invasive potential via protein complex formation. A key example is in hepatocellular carcinoma, where Cu^2+^ acts as a ligand for cluster of differentiation 147 (CD147) to initiate its homomeric aggregation. This event activates the PI3K/AKT signaling axis, setting off a cascade that results in elevated production of matrix metalloproteinases MMP2 and MMP14 [164]. Notably, key components of the copper homeostasis network have emerged as potential biomarkers for metastatic potential. Specifically, the copper chaperone ATOX1 contributes directly to metastasis in cancers like breast cancer by coordinating copper transport along the ATP7A-LOX axis [165]. Furthermore, beyond the well-established LOX axis, copper drives metastasis through direct kinase modulation. Tumor cells alter the expression of copper chaperones ATOX1 and CCS to specifically deliver copper ions to key kinases such as MEK1/2 and ULK1/2. This precise ion delivery drives proliferation, metabolic reprogramming, and metastasis in hepatocellular carcinoma and breast cancer [24].

Additionally, abnormal recombination of iron metabolism is one of the core driving forces for tumor invasion and metastasis [166, 167]. It actively reshapes the extracellular matrix (ECM) and creates a pre-metastatic microenvironment through a multi-level and multi-target mechanism [168]. Its role originates within the cell: as mentioned above, cancer cells accumulate excessive iron through strategies such as overexpressing TfR1, these iron ions catalyze the generation of ROS via the Fenton reaction, leading to sustained oxidative stress [56, 80]. This state of oxidative stress can, on one hand, activate pivotal signaling pathways including NF-κB, in which TfR1 itself physically interacts with the IKK complex to potentiate NF-κB signaling and may further promote the induction of biological programs such as EMT, thereby conferring enhanced migratory and invasive properties to cancer cells [169, 170]. On the other hand, ROS can directly cause lipid peroxidation and damage cell membrane integrity. At the same time, oxidative stress environment may more widely affect protease activity, thereby promoting ECM degradation and remodeling [171]. This process is closely related to the regulation of immune cells in the TME. Iron metabolism significantly affects the polarization of tumor-associated macrophages (TAMs): in the TME, macrophages tend to polarize into the M1 phenotype with pro-inflammatory and anti-tumor potential, characterized by increased expression of iron storage proteins and decreased expression of iron export protein FPN [172]. However, common TAMs in the TME often exhibit a phenotype similar to M2, capable of releasing iron to support tumor growth and immune escape [173]. Furthermore, lipid peroxidation products and the inflammatory milieu resulting from iron overload recruit and affect the function of immunosuppressive cells such as myeloid-derived suppressor cells (MDSCs). These cells suppress T cell activity through mechanisms such as cysteine depletion, thereby creating an immunosuppressive microenvironment locally and providing an "immune-privileged" niche for metastasis [174]. Ferroptosis plays a dual role in this process, where oxidized lipids generated during ferroptosis, such as phosphatidylethanolamines, can act as immunomodulatory signals that recruit and activate antigen-presenting cells, yet may also be exploited by certain immunosuppressive cell populations [175].

**Fig. 4:**
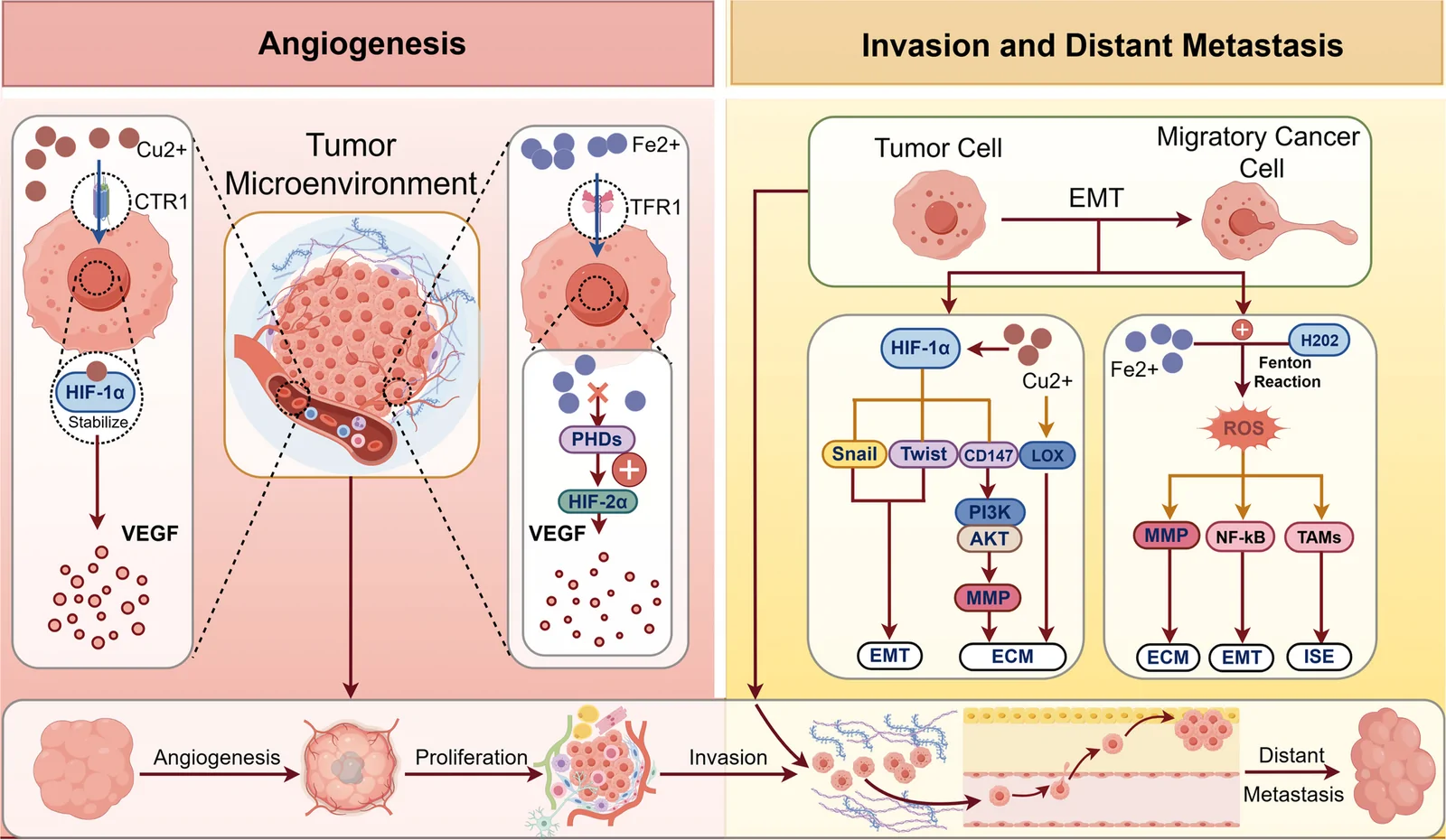
Copper and Iron in Tumor Angiogenesis and Metastasis. *Note.* This figure illustrates the central roles of copper (Cu^2^⁺) and iron (Fe^2^⁺) in driving tumor angiogenesis, invasion, and distant metastasis within the tumor microenvironment (TME).**.** In terms of angiogenesis, copper (via CTR1) and iron (via TFR1) stabilize HIF‑1α and upregulate VEGF, driving endothelial proliferation and tumor angiogenesis. Concerning invasion and metastasis, copper activates HIF‑1α–Snail/Twist to induce EMT and LOX for ECM remodeling, while iron‑generated ROS promotes NF‑κB signaling and immune suppression, collectively enabling tumor cell invasion and distant metastasis. Abbreviations: Cu, Copper; Fe, Iron; HIF-1α, Hypoxia-inducible factor 1-alpha; VEGF, Vascular endothelial growth factor; CTR1, Copper transporter 1; TFR1, Transferrin receptor 1; EMT, Epithelial-mesenchymal transition; ECM, Extracellular matrix; LOX, Lysyl oxidase; PHDs, Prolyl hydroxylases; ROS, Reactive oxygen species; H₂O₂, Hydrogen peroxide; MMPs, Matrix metalloproteinases; TME, Tumor microenvironment

Moreover, iron and copper metabolic networks are closely intertwined. For instance, the copper metabolism protein COMMD10 can modulate HIF1α stability by disrupting copper‑iron homeostasis, thereby influencing radiosensitivity and the ferroptotic process in hepatocellular carcinoma [176]. In summary, iron orchestrates a critical regulatory axis that supports tumor ECM remodeling and metastasis through mediating oxidative stress, modulating immune cell function, and interacting with copper metabolism networks, thereby establishing itself as a highly promising anti-metastatic target. Beyond the classical iron-copper axis, a broader spectrum of trace elements orchestrates invasion and metastasis through distinct pathways. For zinc, tumor cells modulate intracellular zinc flux by altering the expression of zinc transporters such as ZIP6 and ZIP10, thereby influencing the epithelial‑mesenchymal transition process and promoting the invasion and metastasis of cancers such as breast cancer [177]. For selenium, tumor cells reprogram the expression profiles of selenoproteins such as TXNRD, redirecting selenium from fundamental antioxidant defense functions toward pathways that specifically support tumor cell survival and progression during metastatic dissemination [178]. In essence, tumor metastasis is orchestrated by an integrated network of copper, iron, zinc, and selenium, each contributing distinct yet interwoven mechanisms, offering a promising but complex frontier for anti‑metastatic intervention. The convergent pathways through which copper and iron jointly promote angiogenesis, invasion, and metastasis are illustrated in Fig. 4.

## Therapeutic intervention strategies

### Traditional nutritional and pharmacological interventions

Before detailing these strategies, it is critical to emphasize that the vast majority of the interventions discussed below remain at an exploratory, preclinical stage. Except for a few epidemiological associations and early‑phase trials, the mechanistic evidence derives predominantly from in vitro systems, cancer cell lines, and syngeneic or xenograft mouse models. Consequently, these approaches should be interpreted as testable mechanistic hypotheses and experimental prototypes rather than validated clinical paradigms. Our intention is to synthesize current research directions and identify knowledge gaps, not to suggest that any of these strategies are ready for routine clinical application.

#### Nutritional intervention

Nutritional intervention serves as a fundamental strategy for modulating trace element homeostasis. By adjusting dietary intake or supplementing with nutrients, it optimizes elemental levels in the body, thereby influencing the TME and cellular metabolism.

Iron Deprivation Strategy inhibits malignant progression by limiting the bioavailability of iron to tumor cells. Its core mechanisms include the use of iron chelators such as deferoxamine and deferasirox to reduce systemic iron levels, or the application of anti-transferrin receptor 1 antibodies to block tumor-specific iron uptake. Preclinical studies have demonstrated that iron deprivation strategies significantly inhibit tumor proliferation in a variety of cancer models, including liver cancer [179], leukemia (acute myeloid leukemia) [179], lymphoma (diffuse large B-Cell lymphoma) [179], kidney cancer [179], colorectal cancer [13] and prostate cancer [180]. Notably, colorectal cancer tissues often exhibit iron accumulation accompanied by dysregulated expression of iron transporters, which further enhances the theoretical feasibility of iron deprivation strategies in this cancer type [13]. However, systemic iron deprivation may exacerbate tumor-associated anemia, thereby limiting its clinical translation. Future efforts should focus on developing iron metabolism regulators that specifically target the TME to overcome systemic toxicity [180]. Furthermore, combining vitamin C with iron chelators may enhance their antitumor activity through pro-oxidative mechanisms. However, the clinical safety and efficacy of this synergistic effect require systematic validation in prospective trials [179].

The copper chelator tetrathiomolybdate (TTM) reduces copper levels in tumor tissues, inhibits angiogenesis, and has demonstrated antitumor activity in models of breast cancer [181]. A Phase II clinical trial involving patients with high-risk recurrent breast cancer demonstrated that copper depletion using TTM could modulate the TME and exhibited anti-metastatic potential in preclinical models of lung metastasis [24]. For tumors such as esophageal squamous cell carcinoma, zinc supplementation may inhibit proliferation, but excessive intake can promote tumor growth [182]. However, in prostate cancer, the effect is dose‑ and timing‑dependent: > 75 mg/day or > 15 years increases lethal/aggressive risk, whereas low‑dose (1–24 mg/day) post‑diagnosis lowers mortality [183, 184]. Thus, nutritional interventions should be based on evidence strength, and blind supplementation should be avoided.

#### Pharmacological interventions targeting element metabolism

Pharmacological interventions targeting element metabolism involve the use of small-molecule drugs to directly modulate key proteins involved in trace element homeostasis, thereby achieving selective tumor cell killing. By interfering with the uptake, storage, or utilization of essential metals such as iron, copper, and zinc, these agents disrupt metabolic pathways that are critical for tumor proliferation and survival.

Ferroptosis inducers targeting GPX4, such as RSL3 and ML162, or system Xc⁻ inhibitors like erastin, can specifically trigger iron-dependent lipid peroxidation and cell death, showing significant efficacy in cancers such as KRAS-mutant pancreatic cancer and diffuse large B-cell lymphoma [185, 186].

Copper-based therapy includes two major strategies: copper chelators (tetrathiomolybdate, D-penicillamine) reduce bioavailable copper, inhibiting angiogenesis and metastasis [147, 187]; copper ionophores (disulfiram/copper complexes, elesclomol) increase copper concentration in tumor cells, inducing cuproptosis [188, 189]. These can be combined with chemotherapy, radiotherapy, and immunotherapy, and nano-delivery systems are under development [188].

Pharmacological interventions targeting zinc metabolism inhibit tumor growth by modulating pathways associated with zinc homeostasis. The transcription factor E2F4 promotes autophagy by enhancing the transcription of autophagy-related genes ATG2A and ULK2, leading to degradation of metallothioneins and thereby influencing intracellular zinc levels and promoting cancer progression. The deubiquitinating enzyme USP2 stabilizes E2F4 through physical interaction and deubiquitination; therefore, targeting the USP2-E2F4 axis may suppress cancer progression [190]. Zinc oxide nanoparticles have also demonstrated autophagy and apoptosis induction in pancreatic cancer models [191–193]. Additionally, specific selenium compounds such as selenocystine (SeC) can enhance 5-fluorouracil-induced apoptosis in cancer cells by inducing reactive oxygen species-mediated DNA damage and inactivating the ERK pathway [194]. For instance, selenodiazole derivatives act as radiosensitizers by inducing G2/M phase arrest, triggering ROS-mediated DNA damage, and inactivating AKT and MAPKs signaling pathways, thereby significantly enhancing X-ray-induced growth inhibition and clonogenic suppression in melanoma and cervical cancer cells [195].

Moreover, the development of small-molecule inhibitors targeting manganese-dependent enzymes, such as MnSOD, aims to disrupt the adaptation of tumor cells to oxidative stress and their mechanisms of immune evasion. This therapeutic strategy is precisely based on the fact that MnSOD, as a core mitochondrial antioxidant enzyme, whose overexpression represents a key mechanism by which cancer cells resist chemotherapy, radiotherapy, and oxidative damage. Inhibiting MnSOD can disrupt the redox balance in cancer cells, sensitize them to conventional therapies, and potentially induce ferroptosis [7, 196]. The above traditional nutritional and pharmacological intervention strategies are summarized in Table 1.

**Table 1:** Anti-Cancer Strategies Targeting Trace Element Metabolism

Category	Strategy/Drug	Element	Core Mechanism	Development Stage	Representative Cancer Types	References
Nutritional	Iron deprivation (deferoxamine, deferasirox)	Fe	Limit iron bioavailability, inhibit proliferation	Preclinical	Liver cancer, leukemia, colorectal cancer, prostate cancer	[197, 198]
	Copper depletion (tetrathiomolybdate)	Cu	Inhibit angiogenesis & metastasis	Phase II	High-risk recurrent breast cancer	[199, 200]
	Zinc supplementation	Zn	Restore immune surveillance, inhibit proliferation	Preclinical/observational	Esophageal cancer, prostate cancer	[182, 201]
	Selenium supplementation (selenomethionine, Se-yeast)	Se	Enhance antioxidant defense, inhibit angiogenesis	Preclinical/epidemiological	Multiple cancers (prevention)	[202, 203]
Pharmacological	Ferroptosis inducers (RSL3, ML162, erastin)	Fe	Inhibit GPX4/system Xc⁻, lipid peroxidation	Preclinical	KRAS-mutant pancreatic cancer, DLBCL	[204, 205]
	Copper chelators (tetrathiomolybdate, trientine)	Cu	Lower serum Cu, inhibit angiogenesis/metastasis	Preclinical/I/II	Breast cancer, hepatocellular carcinoma	[199, 200, 206]
	Zinc homeostasis modulator (USP2-E2F4 axis)	Zn	Regulate zinc flux, inhibit tumor growth	Preclinical	Cancer progression	[207, 208]
	Selenium compounds (selenocystine, selenodiazole)	Se	ROS-mediated DNA damage, chemosensitization	Preclinical	Melanoma, cervical cancer	[209–211]
	Mn-dependent enzyme inhibitors (MnSOD, arginase)	Mn	Disrupt oxidative stress adaptation, immune evasion	Preclinical	Glioblastoma, cancer immunotherapy	[212–214]
Synergy (chemo/RT)	Iron metabolism sensitization	Fe	Iron chelators + cisplatin/doxorubicin; inhibit Fenton	Preclinical	Multiple solid tumors	[197, 215]
	Copper metabolism sensitization	Cu	Cu ionophores/chelators alleviate hypoxia, enhance RT	Preclinical	Hepatocellular carcinoma	[199, 206, 216]
	Zinc as adjunct to radiotherapy	Zn	Improve taste disorders, oral mucositis	Clinical intervention	Head and neck cancer	[217]
	Selenium radiosensitizers	Se	Induce ROS, disrupt redox balance	Preclinical	Melanoma, cervical cancer	[203, 209]
Synergy (ICI)	Ferroptosis-driven immunogenicity	Fe	Release DAMPs, activate DCs, synergize with α-PD-1	Preclinical	Multiple tumors	[204, 218, 219]
	Copper homeostasis & immune regulation	Cu	Modulate T cells, suppress PD-L1	Preclinical	Cancer immunotherapy	[199, 200, 220]
	Zinc signaling & immune synapse	Zn	Bridge Lck to TCR, enhance T cell activation	Preclinical	Cancer immunotherapy	[179, 201, 207]
	Selenium & immune checkpoint	Se	Promote T cell/NK activity, induce ICD	Preclinical	Cancer immunotherapy	[202, 210, 211]
	Manganese activates cGAS-STING	Mn	Enhance STING pathway, synergize with ICI	Phase I	Advanced metastatic solid tumors	[212, 221, 222]
Interdisciplinary	Multi-element responsive nanoplatforms (Fe/Mn, Zn/Se)	Fe, Mn, Zn, Se	Combine ferroptosis/cuproptosis with STING activation	Preclinical	Hepatocellular carcinoma, colon cancer	[197, 223–225]

*Abbreviations*: *cGAS* cyclic gmp-amp synthase, *DAMPs* damage-associated molecular patterns, *DCs* dendritic cells, *DLBCL* diffuse large b-cell lymphoma, *E2F4* e2f transcription factor 4, *GPX4* glutathione peroxidase 4, *ICI* immune checkpoint inhibitors, *ICD* immunogenic cell death, *KRAS* kirsten rat sarcoma viral oncogene homolog, *Lck* lymphocyte-specific protein tyrosine kinase, *MnSOD* manganese superoxide dismutase, *NK* natural killer, *PD-1* programmed cell death protein 1, *PD-L1* programmed death-ligand 1, *ROS* reactive oxygen species, *RT* radiotherapy, *STING* stimulator of interferon genes, *TCR* t cell receptor, *USP2* ubiquitin-specific protease 2, *Ca* calcium, *Cu* copper, *Fe* iron, *Mn* manganese, *Se* selenium, *Zn* zinc

### Synergistic sensitization strategies based on element metabolism

#### Synergy with chemotherapy/radiotherapy

Iron metabolism sensitization strategies achieve synergistic enhancement with chemotherapy and radiotherapy by interfering with the iron homeostasis of tumor cells. On one hand, iron chelators deplete intracellular iron, directly inhibiting tumor growth while producing synergistic cytotoxic effects with DNA-damaging chemotherapeutic agents such as cisplatin and doxorubicin [13]. Additionally, by suppressing iron-dependent Fenton reactions, they effectively alleviate doxorubicin-induced cardiotoxicity. On the other hand, an iron-deficient environment sensitizes tumor cells to radiotherapy by impairing their antioxidant repair capacity and limiting their Fenton reaction potential, thereby enhancing susceptibility to radiation-induced oxidative damage and achieving radiosensitization [7].

Copper synergizes with chemotherapeutic agents such as cisplatin or radiotherapy primarily through its redox properties to enhance therapeutic efficacy. Copper ions catalyze Fenton-like reactions to generate reactive oxygen species, directly inducing DNA damage and lipid peroxidation, thereby augmenting the cytotoxicity of chemotherapy drugs and overcoming drug resistance [199, 226]. However, the connection between copper metabolism and cisplatin response is bidirectional and complex. The copper efflux transporter ATP7B is well established as a correlate of cisplatin resistance, but this resistance does not reflect active drug efflux. Rather, cisplatin binding to ATP7B perturbs copper homeostasis, and the resultant copper depletion activates secondary detoxification pathways such as glutathione upregulation, indirectly promoting resistance [227]. Shifting the focus to radiotherapy, copper ionophores such as disulfiram/copper complexes or chelators such as tetrathiomolybdate can effectively alleviate tumor hypoxia and enhance radiosensitivity by disrupting hypoxia-inducible factor-1α stability, inducing mitochondrial dysfunction, and promoting immunogenic cell death [206].

Zinc, as an adjunctive therapy, can synergize with chemotherapy or radiotherapy to alleviate side effects and potentially enhance antitumor efficacy. Studies have shown that zinc supplementation has positive effects on radiotherapy-induced taste disorders and oral mucositis, but shows no significant benefit for chemotherapy-related adverse effects. One study demonstrated that zinc supplementation improved taste perception during radiotherapy in patients with head and neck cancer and significantly increased their daily meal intake [228].

The radiosensitizing effect of selenium is primarily mediated through its compounds, such as selenocysteine and methylseleninic acid, which induce excessive reactive oxygen species production in tumor cells, disrupt intracellular redox balance, and cause DNA damage, thereby enhancing the cytotoxic effects of ionizing radiation on cancer cells [195, 202, 229]. Concurrently, selenium downregulates pro-survival signaling pathways such as PI3K/AKT and upregulates pro-apoptotic proteins such as Bax, synergistically promoting radiation-induced apoptosis [230, 231].

#### Synergy with ICIs

Ferroptosis-driven immunogenicity: By inducing ferroptosis in tumor cells, damage-associated molecular patterns (DAMPs) are released, thereby activating antitumor immune responses. Studies have shown that ferroptosis can trigger immunogenic cell death, promote dendritic cell maturation and antigen presentation, and enhance the tumor infiltration and cytotoxic function of CD8^+^ T lymphocytes [232]. Furthermore, ferroptosis exhibits a synergistic effect with ICIs such as anti-PD-1 antibodies, reshaping the tumor immune microenvironment. The combination therapy can significantly inhibit tumor growth and prevent metastasis [233].

Copper homeostasis regulates T-cell function by influencing T-cell metabolism, differentiation, and effector functions, thereby synergizing with ICIs. Studies have shown that copper ions (Cu^2^⁺) play a critical role in T-cell receptor signaling, and disruption of copper homeostasis impairs T-cell activation and proliferation [24]. Furthermore, copper chelators such as tetrathiomolybdate can suppress PD-L1 expression by reducing copper levels in the TME, thereby increasing the number of tumor-infiltrating CD8⁺ T cells and NK cells and enhancing antitumor immune responses [234].

Zinc signaling enhances the immune synapse by modulating intercellular communication among immune cells, thereby potentiating antitumor immune responses. Zinc ions (Zn^2^⁺) play a pivotal role in T-cell receptor signaling, acting as bridging ligands that recruit the kinase Lck into spatial proximity with the TCR, facilitating more efficient signal transduction [235]. Additionally, zinc signaling promotes the activation and differentiation of CD4^+^ and CD8^+^ T cells, and enhances the cytotoxicity of NK cells, thereby synergizing with ICIs to potentiate tumor killing [236].

Selenium enhances the efficacy of ICIs by modulating immune cell function and redox balance. Selenium supplementation promotes T cell proliferation and NK cell activity, and stimulates the production of cytokines such as interferon-gamma [237]. Selenium compounds can induce immunogenic cell death in tumor cells, releasing damage-associated molecular patterns that activate DCs and enhance antigen presentation, thereby synergizing with anti-PD-1 therapy to produce antitumor effects [202].

Manganese significantly enhances antitumor immunity and synergizes with ICIs by activating the cGAS-STING pathway. Manganese ions (Mn^2^⁺) promote dendritic cell maturation and antigen presentation, and activate CD8⁺ T cells and NK cells. In a Phase I clinical trial involving patients with advanced metastatic solid tumors, the combination of Mn^2^⁺ with anti-PD-1 antibodies demonstrated favorable tolerability, induced type I interferon responses, and restored responsiveness to anti-PD-1 therapy in the majority of patients [145].

### Element metabolism in immune engineering

It is important to emphasize that the strategies discussed in this section are supported exclusively by in vitro and preclinical animal studies. At present, not enough clinical evidence is available to validate their therapeutic efficacy or safety in humans. These approaches should therefore be understood as experimental prototypes and conceptual frameworks for future investigation, rather than clinically applicable modalities.

#### Element-triggered nanoplatforms for cancer immunotherapy

Utilizing element concentration gradients unique to the TME as triggering switches to design “smart” nanodelivery systems or prodrugs.

The ferroptosis-driven immune activation strategy achieves synergistic amplification of ferroptosis induction and antitumor immune responses by constructing smart nanoplatforms that specifically respond to high iron concentrations in the TME. A range of iron-based nanomaterials have been developed to exploit the Fenton reaction for inducing lipid peroxidation and ferroptosis, which in turn triggers immunogenic cell death and promotes dendritic cell maturation and T lymphocyte infiltration [238]. Preclinical studies have demonstrated that when these platforms are combined with immune checkpoint inhibitors, enhanced antitumor efficacy and long-term immune memory can be achieved, although these findings remain largely confined to syngeneic mouse models [205, 239, 240].

The copper-dependent drug controlled release strategy achieves synergistic amplification of cuproptosis induction and antitumor immune responses by constructing intelligent nanoplatforms that specifically respond to the highly reducing conditions of the TME [241]. Various copper-based nanoplatforms have been developed to enable targeted delivery of copper ions and immunomodulators, exploiting cuproptosis to trigger immunogenic cell death and reshape the immunosuppressive microenvironment [220]. These platforms have been shown to activate the cGAS-STING signaling pathway, promote dendritic cell maturation and cytotoxic T lymphocyte infiltration in preclinical models [242]. Additionally, copper-based nanomaterials can modulate oxidative stress through Fenton-like reactions, inducing multiple forms of regulated cell death [243].

Zinc ion-based smart nanoplatforms achieve synergistic induction of zinc homeostasis regulation and immunogenic cell death through TME-responsive release mechanisms. Zinc-based nanomaterials, including ZIF-8 and ZnO, have been designed to responsively release Zn^2^⁺ in the acidic TME, triggering tumor cell pyroptosis or apoptosis by disrupting mitochondrial function and inducing oxidative stress [244, 245]. In preclinical studies, these platforms have demonstrated the ability to promote dendritic cell maturation and cytotoxic T lymphocyte infiltration, thereby converting immunologically cold tumors into hot tumors. When combined with immune checkpoint blockade, synergistic antitumor effects have been observed in various cancer models [244]. Furthermore, zinc/manganese-based MOF nanoplatforms have been explored for their dual capacity to induce immunogenic cell death and activate the cGAS-STING pathway [208].

The selenium-based intelligent nanoplatform demonstrates multidimensional application prospects in tumor immunotherapy through its unique redox dual-regulation capability. Selenium exhibits a typical dose-dependent dual-regulation characteristic in tumor immunity: at physiological concentrations, it exerts antioxidant and immune-protective functions through selenoproteins (such as GPx and TXNRD), while at the nanoscale, it can trigger immunogenic cell death by inducing oxidative stress [246]. A variety of selenium-based nanomaterials have been developed to exploit this duality, with some formulations designed to target lymph nodes and activate the cGAS-STING signaling pathway [209]. These platforms have been reported to induce dendritic cell maturation and T cell activation in preclinical models, and have shown the capacity to remodel the tumor immune microenvironment by increasing CD8⁺ T cell infiltration and reducing regulatory T cells [247]. Early-phase clinical exploration of selenium nanoparticles in combination with standard chemotherapy has also been initiated, though these trials remain preliminary and have not yet established definitive survival benefits [248, 249].

Manganese-based smart nanoplatforms exhibit unique advantages in tumor immunotherapy through the dual mechanism of activating the cGAS-STING signaling pathway and inducing immunogenic cell death. Manganese ions, serving as a second messenger for cGAS, can significantly enhance the sensitivity of cGAS to double-stranded DNA and promote the oligomerization of STING protein, thereby inducing the production of type I interferons and pro-inflammatory cytokines to initiate innate immune responses [250, 251]. A range of manganese-based nanoplatforms have been developed to exploit this mechanism, integrating multiple nanozyme activities to enhance oxidative stress and amplify STING pathway activation [212]. These platforms have demonstrated potent immunotherapeutic effects in preclinical models of distant and metastatic tumors. Furthermore, manganese-based nanomedicines have been designed to catalyze the conversion of endogenous H₂O₂ into highly cytotoxic hydroxyl radicals, thereby enhancing chemodynamic therapy and inducing immunogenic cell death while simultaneously acting as cGAS-STING pathway agonists [252, 253]. Early clinical exploration of manganese-based formulations has also been initiated, with preliminary evidence suggesting tolerability and immune activation, though therapeutic efficacy remains to be established in larger controlled trials [254].

The multi-element synergistic response system can integrate the unique functions of two or more elements to achieve synergistic amplification of cell death induction and immune activation. Bimetallic organic framework nanoplatforms, for example, have been designed to simultaneously release iron to mediate the Fenton reaction and induce ferroptosis, while releasing manganese to activate the cGAS-STING pathway [255, 256]. Similarly, copper/zinc cascade response systems have been explored to achieve sequential intervention of immune activation and immunosuppression relief. Zinc/manganese-based MOF nanoplatforms have been shown to activate the cGAS-STING pathway while their enzyme-mimicking activities enhance tumor cell death and induce immunogenic cell death, thereby remodeling the immunosuppressive microenvironment [208].

The element-responsive prodrug strategy leverages the elemental characteristics of the TME to activate prodrugs. For example, chemotherapy prodrugs containing copper-responsive chemical bonds have been designed for specific cleavage and release of the active drug in a high-copper microenvironment. Iron-based metal–organic frameworks have been developed to achieve controlled drug release in the acidic TME, promoting immunogenic cell death and inducing cGAS-STING pathway activation [257]. Additionally, prodrugs have been designed to be specifically cleaved by hydroxyl radicals generated from iron-catalyzed Fenton reactions, achieving spatiotemporal coupling of ferroptosis and chemotherapy [258, 259]. pH/ROS dual-responsive systems have also been explored for cascade-responsive drug release, enabling highly responsive release of chemotherapeutic agents in the high-ROS and acidic environment within tumor cells [258–260]. These studies provide paradigms for constructing cascade-responsive nanoplatforms that enable the sequential release of element chelators and immunomodulators, synergistically remodeling the immune microenvironment. The characteristics and combination strategies of various element-responsive nanoplatforms are presented in Table 2.

**Table 2:** Trace Element Nanoplatforms in Combinatorial Cancer Therapy

Element Category	Nanoplatform	Core Mechanism	Combination Strategy	Cancer Type	Development Stage	References
Fe	Fe-based MOF nanozyme (Quasi Fe MIL-53)	Peroxidase/catalase mimics, GSH depletion, ferroptosis	ICD induction, synergize with anti-PD-1	Colorectal cancer, melanoma	Preclinical	[204]
	ROS-responsive Fe₃O₄ nanoparticles	Charge reversal, enhanced Fe uptake, ferroptosis	Release immunostimulatory peptides, combine with α-PD-L1	Lung metastatic tumors	Preclinical	[205]
	Fe-based metal–organic framework (MIL-100)	Ferroptosis induction, iron homeostasis disruption	Chemo-immunotherapy, combination with doxorubicin	Hepatocellular carcinoma	Preclinical	[261]
Cu	NK cell membrane-camouflaged Cu-BODIPY	Dual-wavelength photothermal, ROS/GSH depletion, cuproptosis	Photothermal therapy + ICD + immune checkpoint blockade	Breast cancer	Preclinical	[220]
	Macrophage membrane-coated Cu-MOF encapsulating elesclomol	H₂S-responsive photothermal, Cu ion release, cuproptosis	Photothermal therapy + ICD + DAMP release	Colon cancer	Preclinical	[262]
	Defective Cu-HITP nanozyme with unsaturated Cu–N2 sites	ROS generation, cuproptosis, MerTK inhibition	Innate immune-checkpoint blockade + Mn2 + STING activation	Multiple solid tumors	Preclinical	[263]
Zn	ZIF-8 encapsulating PD-L1 siRNA	Acid-triggered Zn²⁺ release, pyroptosis	Pyroptosis + PD-L1 silencing + immune checkpoint blockade	Breast cancer, melanoma	Preclinical	[207]
	Zn/Mn-MOF (AMP@Zn/Mn-MOF)	Peroxidase/GPx mimetic, cGAS-STING activation	ICD + STING activation + αPD-L1 synergy	Colon cancer	Preclinical	[208]
	PYT@ZIF8@siRNA nanoparticles	Zn overload, mitophagy, disrupt bacterial membrane	Chemo + anti-PD-1 synergy, dual elimination of tumor and bacteria	Oral squamous cell carcinoma	Preclinical	[264]
	ZnO/Pheophorbide-a nanoflakes	Photobiomodulation, antioxidant, anti-inflammatory	Photobiomodulation therapy + neurovascular repair	Spinal cord injury (glioma)	Preclinical	[264]
Se	Bifunctional peptide-Se nanocomposite (SA)	Chaperone-mediated autophagy, lysosomal PD-L1 degradation	PD-L1 degradation + T cell activation	Colorectal cancer	Preclinical	[210]
	Se nanoparticles loaded with MDM2-targeting peptide MI	p53 reactivation, immune microenvironment remodeling	p53 reactivation + immune checkpoint modulation	Colorectal cancer	Preclinical	[211]
	Selenium nanoparticles (broad-spectrum)	Redox modulation, selenoprotein synthesis	Radiosensitization + immune activation	Multiple cancers	Preclinical/Clinical	[246]
Mn	Multifunctional Mn-based nanozyme (MnOxChP)	Nanozyme activities + cholesterol oxidase, cholesterol depletion	STING activation + ICD	Metastatic tumors	Preclinical	[212]
	TME-responsive Mn-nanomedicine (LMMH)	Release Mn²⁺ + glycolysis inhibitor LND, CDT	Chemodynamic therapy + cGAS-STING activation	Primary & metastatic tumors	Preclinical	[214]
	Dual-mode immune nanoactivator (Mn²⁺ + quercetin)	NIR-accelerated release, ROS, DNA damage	cGAS-STING activation + ICD + anti-CTLA-4 synergy	Melanoma	Preclinical	[213]
	Ultrasound-responsive Mn/Se nanozyme	US-triggered PANoptosis, STING activation	Sonodynamic therapy + ICD + PD-1 blockade	Bladder cancer	Preclinical	[222]
	Mn-based nanoactivators	cGAS-STING activation, innate immunity enhancement	STING activation + immune checkpoint blockade	Multiple solid tumors	Preclinical/Phase I	[265, 266]
	Mn-induced ferroptosis nanoplatform	Type-I IFN dependent inhibition of DHODH	Ferroptosis + innate immunity	Multiple cancers	Preclinical	[267]
	Mn2 +/polydopamine hollow organosilica nanoreactors	ROS cascade amplification, RNS storm	Oxidative stress + ICD + immunotherapy	Breast cancer	Preclinical	[268]
Zn/Se	ZnS/Se/BSA nanoclusters (ZSB NCs)	Lymph node targeting, release Zn [113]⁺ & selenoproteins, cGAS-STING activation	Dual ETE-mediated immunotherapy + DC/T cell activation	Breast cancer	Preclinical	[209]
Zn/Mn	Biomimetic nanoplatform (hollow ZIF-8 + MSC membrane)	Acidic TME-responsive release of polyphyllin II, ferroptosis, mtDNA release	Ferroptosis + cGAS-STING activation + M2 → M1 repolarization	Hepatocellular carcinoma	Preclinical	[255]
Fe/Mn	Fe/Mn bimetallic MOF	Fenton reaction + cGAS-STING activation	Ferroptosis + STING pathway activation	Hepatocellular carcinoma	Preclinical	[255, 261]

*Abbreviations*: *cGAS* cyclic gmp-amp synthase, *CDT* chemodynamic therapy, *CTLA-4* cytotoxic t-lymphocyte-associated protein 4, *DAMP* damage-associated molecular pattern, *DC* dendritic cell, *DHODH* dihydroorotate dehydrogenase, *GPx* glutathione peroxidase, *GSH* glutathione, *ICD* immunogenic cell death, *IFN* interferon, *LND* lonidamine, *MerTK* mer tyrosine kinase, *MOF* metal–organic framework; *MSC* mesenchymal stem cell, *mtDNA* mitochondrial dna, *NIR* near-infrared, *NK* natural killer, *PANoptosis* panoptosis, *PD-1* programmed cell death protein 1, *PD-L1* programmed death-ligand 1, *RNS* reactive nitrogen species, *ROS* reactive oxygen species, *SA* bifunctional peptide-selenium nanocomposite, *SeNPs* selenium nanoparticles, *siRNA* small interfering rna, *STING* stimulator of interferon genes, *TLR* toll-like receptor, *US* ultrasound, *ZSB* NCs, zns/se/bsa nanoclusters, *Cu* copper, *Fe* iron, *Mn* manganese, *Se* selenium, *Zn* zinc

#### Engineering immune cells to sense elemental signals

Through genetic engineering of adoptive immune cells, endowing them with the ability to sense specific elemental signals and mount enhanced responses can significantly improve their survival and functional persistence within the immunosuppressive TME. Researchers have developed a computationally designed synthetic receptor platform capable of creating artificial receptors that detect soluble signals in the TME and convert them into co-stimulatory signals. These synthetic receptors, known as TME-sensing switch receptors, can be combined with chimeric antigen receptor T cells to significantly enhance anti-tumor efficacy [269]. By leveraging synthetic biology principles to couple element-responsive elements with effector genes, gene expression systems can be constructed that automatically turn on or off under specific elemental concentrations. For instance, an iron-sensitive enhancement circuit introduces an iron-responsive element-controlled gene expression cassette into CAR-T cells, enabling them to automatically activate the expression of cytokines that promote T cell survival or effector molecules that resist exhaustion upon infiltrating iron-rich tumor regions, thereby acquiring enhanced adaptability and persistence [270]. The hypoxia/low zinc dual-controlled switch simultaneously senses the hypoxic and low-zinc state in the tumor core, driving therapeutic gene expression only when both conditions are met, thereby achieving more precise spatiotemporal control and avoiding off-target toxicity to normal tissues [271].

Equipping immune cells with exogenous element metabolism-related proteins can enhance their ability to resist oxidative stress and elemental imbalance in the TME. Strategies for developing element-metabolism-armored cells include overexpressing selenoproteins such as GPX4 or thioredoxin reductase in CAR-T cells to scavenge excessive reactive oxygen species and prevent ferroptosis or apoptosis. Simultaneously, introducing the iron export pump FPN1 helps avoid mitochondrial dysfunction and lipid peroxidation damage caused by an iron-rich environment [255]. Research indicates that a biomimetic nanoplatform constructed by coating mesenchymal stem cell membranes onto hollow ZIF-8 MOFs can release polyphyllin II in response to the acidic TME. This induces ferroptosis in hepatocellular carcinoma cells, triggers mitochondrial stress leading to the release of endogenous mitochondrial DNA, and synergizes with Zn^2^⁺ to specifically activate the cGAS-STING pathway. This drives dendritic cell maturation and promotes the repolarization of TAMs from the M2 to the M1 phenotype, thereby significantly inhibiting the progression of hepatocellular carcinoma [272]. Additionally, researchers have converted the extracellular domains of natural cytokine receptors into modular extracellular sensor architecture receptors, generating a variety of functional and orthogonal synthetic cytokine receptors. These can be used to engineer T cells to sense immunosuppressive signals and respond with tailored transcriptional outputs, thereby supporting CAR-T cell activity [273].

Utilizing the spatial heterogeneity of elemental concentrations within the TME, immune cells can be engineered to actively migrate along elemental concentration gradients toward the tumor core. Iron-signal-guided tumor homing leverages the high iron concentration characteristic of tumor regions by designing chimeric antigen receptors sensitive to conformational changes induced by iron ions. This enables immune cells to recognize and migrate to iron-rich tumor tissues while simultaneously triggering killing activation through receptor cross-linking [274]. Researchers have also developed an engineered bacterium-driven biohybrid system capable of achieving precise tumor-targeted delivery of STING agonists through synthetic biology approaches. By leveraging the hypoxia-tropic characteristics of the bacterial strain, the system selectively accumulates in tumor regions and undergoes responsive dissociation, releasing copper ions to induce copper overload-mediated cuproptosis. Simultaneously, laser irradiation enables spatiotemporal control over the release of STING agonists, ensuring precise delivery to the tumor site [275].

Designing intracellular signaling modules that are activated only in the presence of specific elements can prevent systemic toxicity caused by non-specific activation of immune cells. A zinc-ion-activated killing enhancement module utilizes zinc finger protein domains as intracellular sensors. When immune cells are activated accompanied by zinc influx, the conformation of the zinc finger protein changes, initiating the expression of effector molecules or co-stimulatory molecules that enhance cytotoxicity, thereby achieving conditional enhancement of killing function [276]. The copper-ion-responsive immune checkpoint blockade system introduces a PD-1 expression silencing element regulated by copper ion concentration into T cells. When T cells infiltrate copper-rich tumor regions, they automatically downregulate PD-1 expression, relieving immune checkpoint inhibition and enhancing local anti-tumor responses. This strategy, combining bacterial therapy with elemental metabolism regulation, provides new insights into engineering immune cell sensing systems [275].

#### Element-releasing scaffolds for local immune engineering

Creating a microenvironment with sustainable and regulatable element release at the tumor site using functional biomaterials can guide immune cell behavior and reshape anti-tumor immune responses, being particularly suitable for preventing local recurrence after surgery and local treatment of unresectable tumors. Researchers have developed hyaluronic acid-coated nanoparticles loaded with irinotecan and resiquimod based on the MIL-100(Fe) metal–organic framework. These nanoparticles can achieve controlled drug release in the acidic TME, promote immunogenic cell death, induce the activation of the cGAS-STING pathway, and enhance immune cell function. When combined with anti-PD-L1 therapy, they can significantly strengthen anti-tumor immunity, especially exerting a remarkable tumor-suppressive effect on distant tumors [257]. Zinc-doped biodegradable scaffolds release Zn^2^⁺, which can not only directly induce apoptosis of residual tumor cells but also synergistically eliminate micrometastases by activating DCs and enhancing CD8⁺ T cell function [255].

Loading element chelators or ionophores into injectable hydrogels enables local, sustained, and controllable regulation of element homeostasis. Studies have shown that a biomimetic nanoplatform constructed by coating mesenchymal stem cell membranes on hollow ZIF-8 MOFs can acid-responsively release polyphyllin Ⅱ in the TME, induce ferroptosis in hepatocellular carcinoma cells, trigger mitochondrial stress leading to the release of endogenous mitochondrial DNA, and synergize with Zn^2^⁺ to specifically activate the cGAS-STING pathway. This drives the maturation of DCs and promotes the repolarization of TAMs from the M2 to M1 phenotype, thereby significantly inhibiting hepatocellular carcinoma progression [255]. After loading the iron chelator deferoxamine, the thermosensitive chitosan/gelatin hydrogel can be locally injected following tumor resection. It enables sustained release of deferoxamine for more than 2 weeks, effectively reducing local iron levels, inhibiting the growth of micrometastases, while avoiding the risk of anemia caused by systemic iron deprivation [258, 259]. Dual-element synergistic release systems, such as hyaluronic acid hydrogels co-loaded with Zn^2^⁺ and Mn^2^⁺, enhance the activity of local immune cells via Zn^2^⁺ and activate the cGAS-STING pathway through Mn^2^⁺. They synergistically remodel the immunosuppressive microenvironment, significantly prolonging survival in mouse breast cancer models [208]. Studies have also developed pH/ROS dual-responsive nanoparticles loaded with docetaxel. Utilizing cinnamaldehyde to self-supply ROS during mitochondrial damage, these nanoparticles accelerate the degradation of ROS-responsive moieties and promote docetaxel release, significantly inhibiting tumor metastasis and enhancing the efficacy of anti-PD-1 antibodies [258]. Cyclic RGD-functionalized pH/ROS dual-responsive nanoparticles promote deep tumor tissue penetration and cancer cell-specific targeting via cRGD. They release cinnamaldehyde in the acidic environment of lysosomes, while high intracellular ROS levels in tumor cells induce nanoparticle dissociation to achieve complete drug release, significantly enhancing the anti-tumor effect [259].

Combined with the elemental metabolic profile of patients’ tumor tissues, 3D-printed biomimetic scaffolds containing gradient-concentration elemental modulators enable personalized precision therapy. Studies have shown that 3D-printed hybrid hydrogel scaffolds loaded with doxorubicin achieve controlled drug release and effectively prevent postoperative tumor recurrence. Meanwhile, rat bone marrow mesenchymal stem cells loaded in the scaffolds exhibit restored cell viability within a 14-day culture period, providing structural support for postoperative tissue repair [277]. Porous scaffolds fabricated by low-temperature 3D printing can deliver two-dimensional black phosphorus nanosheets, doxorubicin hydrochloride, and osteogenic peptide in situ.Under near-infrared laser irradiation, the photothermal effect of black phosphorus nanosheets triggers drug release to achieve synergistic photothermal-chemotherapy.Meanwhile, black phosphorus nanosheets can significantly alleviate the long-term toxicity of released doxorubicin during bone regeneration in vivo [278]. The biphasic hydrogel scaffold consists of two types of core/shell fibers for drug loading and stem cell loading, respectively.Black phosphorus nanosheets incorporated in the shell of drug-loaded fibers confer the scaffold with near-infrared photothermal effect, which triggers drug release to achieve synergistic photothermal-chemotherapy.The shell of stem cell-loaded fibers provides protection to maintain cell viability and promotes soft tissue regeneration in vivo [279]. After analyzing the elemental distribution characteristics in tumor tissues and their surroundings using ICP-MS or synchrotron radiation X-ray fluorescence imaging, degradable scaffolds with corresponding elemental concentration gradients can be designed based on these data for postoperative implantation to reshape local elemental homeostasis [256, 257]. Multi-zone gradient scaffolds can construct regions with high concentrations of Fe^3^⁺/Cu^2^⁺ and regions with high concentrations of Zn^2^/Mn^2^⁺ within the same scaffold. On the side adjacent to the tumor, they release high levels of iron or copper ions to induce ferroptosis or cuproptosis in residual tumor cells and release antigens. On the side adjacent to normal tissue, they release zinc or manganese ions to promote the activation and proliferation of infiltrating immune cells, achieving an integrated approach to both killing and activation [208].

**Fig. 5:**
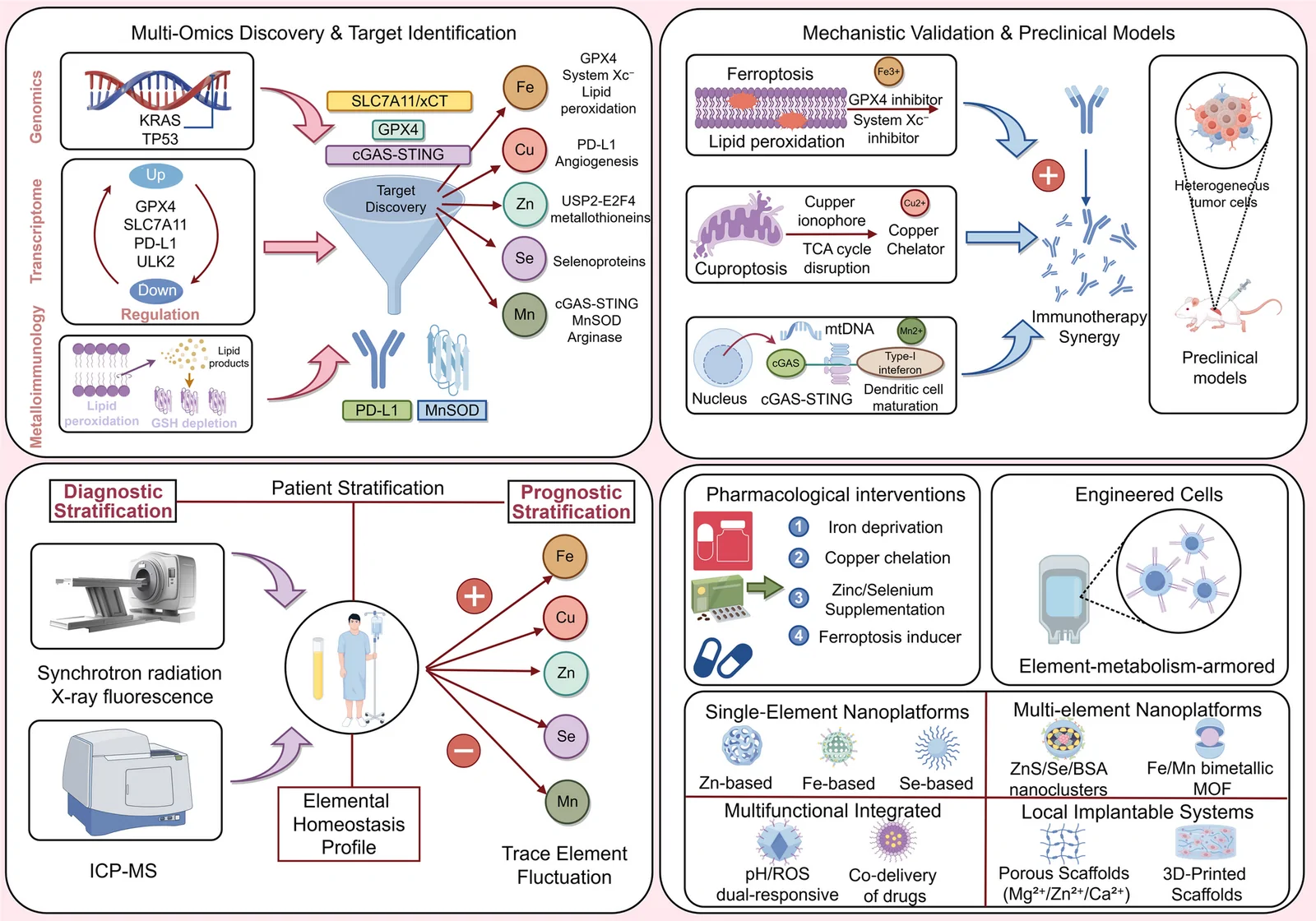
Integrated framework linking multi-omics discovery, mechanistic validation, and translational strategies centered on elemental homeostasis. Multi-omics approaches (genomics, metabolism, pathology) identify key regulators including KRAS, TP53, GPX4, SLC7A11 (system Xc⁻), PD-L1, and ULK2, along with glutathione (GSH) depletion, establishing ferroptosis and immune modulation as core mechanisms. Mechanistic validation focuses on ferroptosis (GPX4 inhibition, lipid peroxidation), copper toxicity (TCA cycle disruption, mtDNA damage, cGAS-STING activation), and immune synergy, with trace elements (Zn, Se, Mn) functioning through metallothioneins, selenoproteins, and MnSOD. Diagnostic stratification employs synchrotron radiation X-ray fluorescence (SR-XRF) for high-resolution elemental imaging and inductively coupled plasma mass spectrometry (ICP-MS) for quantitative analysis, enabling patient and prognostic stratification based on elemental homeostasis and trace element fluctuations. Pharmacological interventions include iron deprivation, copper chelation, Zn/Se supplementation, and ferroptosis inducers, which are further developed into single-element nanoplatforms (Zn-based, Fe-based, Se-based) and multifunctional integrated systems such as pH/ROS dual-responsive carriers, co-delivery platforms, and 3D-printed porous scaffolds. All evidence supporting the strategies depicted in this figure derives from in vitro and preclinical animal models. Clinical validation in humans has not yet been established. Abbreviations: GPX4, Glutathione Peroxidase 4; SLC7A11, Solute Carrier Family 7 Member 11; PD-L1, Programmed Death-Ligand 1; ULK2, Unc-51 Like Autophagy Activating Kinase 2; GSH, Glutathione; mtDNA, Mitochondrial DNA; cGAS-STING, Cyclic GMP-AMP Synthase-Stimulator of Interferon Genes; MnSOD, Manganese Superoxide Dismutase; SR-XRF, Synchrotron Radiation X-ray Fluorescence; ICP-MS, Inductively Coupled Plasma Mass Spectrometry; ROS, Reactive Oxygen Species; Zn^2^⁺, Zinc ion

The design of smart hydrogels responsive to TME characteristics enables the programmed release of immunomodulators. An ROS-responsive hydrogel loads the ferroptosis inducer RSL3 into a polyethylene glycol hydrogel containing thioether bonds. Under the high ROS conditions in the local tumor environment, the bonds are specifically cleaved to release RSL3, triggering ferroptosis in residual tumor cells and releasing damage-associated molecular patterns. Simultaneously, the degradation products of the hydrogel can act as adjuvants to enhance antigen presentation [258, 260]. pH or metal ion dual-responsive release systems, such as peptide hydrogels containing histidine residues, undergo conformational changes in the acidic and high Fe^2^⁺ or high Cu^2^⁺ TME. This enables the controlled release of STING agonists or TLR agonists, synergistically activating anti-tumor immunity alongside metal ion-induced cell death [260]. Researchers have developed an ROS/pH dual-responsive nanodrug delivery system based on MXene (Ti₃C₂). Doxorubicin is conjugated to the surface of MXene via an ROS-cleavable thioketal linkage and coated with pH-responsive polydopamine as a capping molecule. This system combines efficient photothermal conversion performance with good biocompatibility, enabling highly responsive release of doxorubicin in the high-ROS and acidic environment within tumor cells [260]. MOFs have shown great potential as platforms for activating the cGAS-STING pathway in tumor immunotherapy. They can serve as delivery vehicles for agonists or directly act as agonists themselves, combining multimodal strategies to enhance anti-tumor immune responses through the cGAS-STING pathway [256]. The engineered zinc/manganese-based MOF nanoplatform not only significantly activates the cGAS-STING pathway to promote innate immune responses, but its peroxidase and glutathione oxidase-mimicking activities also enhance tumor cell death, effectively inducing immunogenic cell death. This amplifies cGAS-STING pathway activation and remodels the immunosuppressive TME. When combined with αPD-L1, it produces a significant abscopal effect, thus inhibiting the growth of distant tumors (see Fig. 5).

In summary, while trace element‑based therapeutic strategies are experimentally promising, their clinical translation is hindered by sparse survival evidence, substantial biological and pharmacological barriers, and the premature nature of the "platform" concept, necessitating rigorous biomarker‑guided trials before any meaningful patient benefit can be established.

## Conclusive remarks

Trace elements intricately orchestrate cellular life-and-death decisions, metabolic fate, and immune responses through a complex and highly interconnected network. This review systematically elucidates the dysregulation of trace element homeostasis as a core driver of tumor initiation and progression, revealing the molecular basis by which tumor cells hijack the body's elemental resources through an "elemental addiction" mechanism. Furthermore, it delves into the substantial potential of emerging cell death pathways, such as ferroptosis and cuproptosis, in cancer therapy [280, 281]. From systemic elemental abnormalities at the circulatory level to local metabolic reprogramming in the tumor, the dual roles of trace elements permeate the entire process of tumor evolution, providing a rich biomarker repository for cancer risk warning, early diagnosis, and precision intervention [281].

However, a note of caution is warranted. As the preceding chapters have made clear, our current understanding of the role of individual trace elements in cancer biology remains remarkably limited. For most elements, fundamental questions persist: whether supplementation or reduction of intake and systemic levels would be health‑promoting, what constitutes the optimal concentration window, and how these windows vary across different cancer types, stages, and patient populations. The vast majority of mechanistic evidence derives from in vitro systems and preclinical animal models, and clinical validation is sparse. Without a solid and comprehensive database addressing these basic issues, speculation on multi‑element platforms or personalized element‑based therapy remains premature. These limitations should not be viewed as discouraging, but rather as defining the essential research priorities for the field.

Going forward, future investigations should prioritize establishing robust foundational knowledge: systematic profiling of trace element concentrations in human tissues across cancer types, longitudinal studies to clarify causal relationships between elemental status and clinical outcomes, and rigorous pharmacokinetic and pharmacodynamic studies of element‑based interventions. Only when such a database is in place can the field responsibly move toward the rational design of element‑targeted therapeutic strategies. Until then, the perspectives offered in this review should be understood as heuristic frameworks for stimulating hypothesis‑driven research, rather than as imminent clinical realities.

## Supplementary Information

Supplementary Material 1.

## Data Availability

No datasets were generated or analysed during the current study.

## Abbreviations

ACOD1: Aconitate decarboxylase 1
ACSL4: Acyl-coenzyme A synthetase long-chain family member 4
AMPK: AMP-activated protein kinase
ANG: Angiogenin
ATG2A: Autophagy-related 2A
ATG4B: Autophagy-related 4B
ATOX1: Antioxidant protein 1
ATP7A: Copper-transporting ATPase 7A
ATP7B: Copper-transporting ATPase 7B
Bax: Bcl-2-associated X protein
CAR-T: Chimeric antigen receptor T cells
CCS: Copper chaperone for superoxide dismutase
CD147: Cluster of differentiation 147
CD163: Cluster of differentiation 163
CDT: Chemodynamic therapy
cGAS: Cyclic GMP-AMP synthase
COMMD10: Copper metabolism MURR1 domain containing 10
COX: Cytochrome c oxidase
COX-2: Cyclooxygenase-2
COX17: Cytochrome c oxidase copper chaperone 17
CP: Ceruloplasmin
cRGD: Cyclic arginine-glycine-aspartic acid peptide
CTLA-4: Cytotoxic T-lymphocyte-associated protein 4
CTR1: Copper transporter 1
Cu: Copper
DAMPs: Damage-associated molecular patterns
DCs: Dendritic cells
DcytB: Duodenal cytochrome B
DHODH: Dihydroorotate dehydrogenase
DLAT: Dihydrolipoamide S-acetyltransferase
DMT1: Divalent metal transporter 1
E2F4: E2F transcription factor 4
ECM: Extracellular matrix
EMT: Epithelial-mesenchymal transition
ER: Estrogen receptor
ERK: Extracellular signal-regulated kinase
ESCC: Esophageal squamous cell carcinoma
FDX1: Ferredoxin 1
Fe: Iron
FGF2: Fibroblast growth factor 2
FPN1: Ferroportin 1
GM-CSF: Granulocyte-macrophage colony-stimulating factor
GPX: Glutathione peroxidase
GPX4: Glutathione peroxidase 4
GSH: Glutathione
HIF-1α: Hypoxia-inducible factor 1α
HIF-2α: Hypoxia-inducible factor 2α
HIFs: Hypoxia-inducible factors
HO-1: Heme oxygenase-1
ICIs: Immune checkpoint inhibitors
ICP-MS: Inductively coupled plasma mass spectrometry
IFN: Interferon
IKK: IκB kinase
IL-12: Interleukin-12
JNK: C-Jun N-terminal kinase
KRAS: Kirsten rat sarcoma viral oncogene homolog
LC3: Microtubule-associated protein 1A/1B-light chain 3
Lck: Lymphocyte-specific protein tyrosine kinase
LND: Lonidamine
LOX: Lysyl oxidase
LOXL: Lysyl oxidase-like
LPCAT3: Lysophosphatidylcholine acyltransferase 3
MAPK: Mitogen-activated protein kinase
MDSCs: Myeloid-derived suppressor cells
MEK1: Mitogen-activated protein kinase kinase 1
MerTK: Mer tyrosine kinase
MHC II: Major histocompatibility complex class II
MMP-2: Matrix metalloproteinase-2
MMP-9: Matrix metalloproteinase-9
Mn: Manganese
MnSOD: Manganese superoxide dismutase
MOFs: Metal-organic frameworks
MSC: Mesenchymal stem cell
MT: metallothionein
mtDNA: mitochondrial DNA
mTOR: mechanistic target of rapamycin
NF-κB: nuclear factor-κB
NIR: near-infrared
NK cells: natural killer cells
Nrf2: nuclear factor erythroid 2-related factor 2
PD-1: programmed cell death protein 1
PD-L1: programmed death-ligand 1
PE: phosphatidylethanolamine
PHDs: prolyl hydroxylases
PI3K: phosphoinositide 3-kinase
PTGS2: prostaglandin-endoperoxide synthase 2
PUFA: polyunsaturated fatty acid
RNS: reactive nitrogen species
ROS: reactive oxygen species
SA: bifunctional peptide-selenium nanocomposite
Se: selenium
SeC: selenocystine
SELENOP: selenoprotein P
SeNPs: selenium nanoparticles
siRNA: small interfering RNA
SLC7A11: solute carrier family 7 member 11
SOD: superoxide dismutase
SOD1: superoxide dismutase 1
SOD2: superoxide dismutase 2
SR-XRF: synchrotron radiation X-ray fluorescence
STEAP: six-transmembrane epithelial antigen of the prostate
STING: stimulator of interferon genes
System Xc⁻: cystine/glutamate antiporter
TAK1: transforming growth factor beta-activated kinase 1
TAMs: tumor-associated macrophages
TCA: tricarboxylic acid
TCR: T-cell receptor
Tf: transferrin
TfR1: transferrin receptor 1
TGN: trans-Golgi network
TLR: toll-like receptor
TME: tumor microenvironment
TP53: tumor protein p53
TTM: tetrathiomolybdate
TXNRD: thioredoxin reductase
ULK2: Unc-51 like autophagy activating kinase 2
uPA: urokinase-type plasminogen activator
US: ultrasound
USP2: ubiquitin-specific protease 2
VEGF: vascular endothelial growth factor
VEGFR2: vascular endothelial growth factor receptor 2
WHO: World Health Organization
ZIF-8: zeolitic imidazolate framework-8
ZIP: Zrt/Irt-like protein (zinc transporter family)
Zn: zinc
ZnT: zinc transporter (SLC30A family)
ZSB NCs: ZnS/Se/BSA nanoclusters
