# Supplementary material for: Trace elements and tumors: homeostatic networks, molecular mechanisms and new horizons for precision intervention
Source: Mol Cancer. 2026 Jul 30;25:255. doi: 10.1186/s12943-026-02754-y (PMC13711705; doi:10.1186/s12943-026-02754-y)
Supplement: Supplementary file 1 [file 12943_2026_2754_MOESM1_ESM.docx]

| **Cancer Type** | **Element** | **Study design (n)** | **Geographical location** | **Population** | **Baseline levels** | **Key Findings** | **Reference** |
| --- | --- | --- | --- | --- | --- | --- | --- |
| Esophageal Cancer | Zn (tissue concentration) | Prospective observational study (n=132; 60 cases, 72 controls) | Linzhou (formerly Linxian), People's Republic of China | Residents of Linzhou, China, who underwent endoscopy with biopsy in 1985 as part of the baseline evaluation for the Dysplasia Trial, with 16 years of follow-up | Median esophageal tissue zinc concentration: 44 ng/cm² in cases vs. 57 ng/cm² in controls | High tissue zinc concentration was strongly associated with a reduced risk of developing esophageal squamous cell carcinoma | ^1^ |
| Esophageal Cancer | Zn (whole blood concentration) | Case-control study (150 cases, 177 controls) | Chaoshan region, southeastern China | ESCC pathologically confirmed at Cancer Hospital of Shantou University Medical College (2001–2020) | whole blood, median [IQR], μg/L): Cases = 5,918.15 (5,012.34–6,709.62); Controls = 5,853.65 (4,963.44–6,754.92) | Zn showed a U-shaped association with ESCC risk | ^52^ |
| Esophageal Cancer | Zn (serum concentration) | Case-control study (185 cases, 191 controls) | Southern China (Shantou area) | ESCC patients from the Cancer Hospital of Shantou University Medical College (age 40–85) | (serum, median [IQR], μg/L): Cases = 670.13 (527.95–889.97); Controls = 834.78 (633.38–1,052.74) | Zn was significantly inversely associated with ESCC risk | ^74^ |
| Esophageal Cancer | Zn (dietary intake) | Prospective cohort study (n=47,405; 201 ESCC cases) | Golestan Province, northeastern Iran (high-risk region for ESCC) | Healthy adults aged 40–75 years enrolled in 2004–2008, followed through 2014 | Median dietary zinc intake (by quartile): Q1 = 6.8 mg/d, Q2 = 8.9 mg/d, Q3 = 10.5 mg/d, Q4 = 12.9 mg/d | Inverse association in continuous model (HR per 1 mg/d = 0.87; 95% CI: 0.77–0.98; P=0.027), but quartile trend not significant (Q4 vs Q1 HR=0.56; 95% CI: 0.28–1.12; P-trend=0.097). | ^26^ |
| Esophageal Cancer | Zn (dietary intake) | Systematic review and dose-response meta-analysis of observational studies (20 articles; 11 studies on zinc; 4,855 cases from 1,387,482 participants) | Global (included studies from Asia, the United States, and Europe) | General adult populations from cohort and case-control studies | Dietary zinc intake (range across studies); dose-response analysis per 5 mg/day increase | Each 5 mg/day increase in dietary zinc intake was associated with a 15% reduction in esophageal cancer risk (OR = 0.85, 95% CI: 0.77–0.93), with a significant inverse association observed in cohort studies. | ^40^ |
| Esophageal Cancer | Cu (whole blood concentration) | Case-control study (150 cases, 177 controls) | Chaoshan region, southeastern China | ESCC pathologically confirmed at Cancer Hospital of Shantou University Medical College (2001–2020) | Whole blood, median [IQR], μg/L: Cases = 886.52 (775.90–1,027.64); Controls = 844.83 (715.43–976.30) | High Cu was positively associated with ESCC risk | ^52^ |
| Esophageal Cancer | Cu (dietary intake) | Prospective cohort study (n=47,405; 201 ESCC cases) | Golestan Province, northeastern Iran (high-risk region for ESCC) | Healthy adults aged 40–75 years enrolled in 2004–2008, followed through 2014 | Median dietary copper intake (by quartile): Q1 = 1.1 mg/d, Q2 = 1.4 mg/d, Q3 = 1.7 mg/d, Q4 = 2.1 mg/d | A nonlinear association was observed between dietary copper intake and ESCC risk (P-nonlinear = 0.029), but no clear linear trend was found (Q4 vs. Q1 HR = 0.67, 95% CI: 0.53, 1.30; P-trend = 0.20). | ^26^ |
| Esophageal Cancer | Cu (dietary intake) | Systematic review and dose-response meta-analysis of observational studies (20 articles; 4 studies on copper; 4,855 cases from 1,387,482 participants) | Global | General adult populations from cohort and case-control studies | Dietary copper intake (range across studies); dose-response analysis per 1 mg/day increase | No significant association was found between dietary copper intake and esophageal cancer risk (highest vs. lowest category OR = 1.13, 95% CI: 0.51–2.50; dose-response OR per 1 mg/day = 1.00, 95% CI: 0.79–1.28). | ^40^ |
| Esophageal Cancer | Fe (dietary intake) | Systematic review and dose-response meta-analysis of observational studies (20 articles; 13 studies on total iron; 4,855 cases from 1,387,482 participants) | Global (included studies from Asia, the United States, and Europe) | General adult populations from cohort and case-control studies | Dietary total iron intake (range across studies, not a single median); dose-response analysis per 5 mg/day increase | Each 5 mg/day increase in dietary total iron intake was associated with a 15% reduction in esophageal cancer risk (OR = 0.85, 95% CI: 0.79–0.92), with a stronger inverse association observed in Asian populations and for ESCC. | ^40^ |
| Esophageal Cancer | Se (whole blood concentration) | Case-control study (150 cases, 177 controls) | Chaoshan region, southeastern China | ESCC pathologically confirmed at Cancer Hospital of Shantou University Medical College (2001–2020) | Whole blood, median [IQR], μg/L): Cases = 166.53 (127.65–194.35); Controls = 189.56 (158.27–229.82) | Se showed a linear inverse association with ESCC risk | ^52^ |
| Esophageal Cancer | Se (serum concentration) | Prospective nested cohort study (n=1,103 randomly selected from 29,584 participants; 75 ESCC deaths during 15-year follow-up) | Linxian, Henan Province, China (high-risk region for ESCC and gastric cardia cancer) | Healthy adults aged 40–69 years enrolled in the General Population Trial of the Nutrition Intervention Trials, with blood drawn in 1985 and followed through 2001 | Mean serum selenium = 0.93 μmol/L (73 μg/L); 69% of subjects were selenium deficient (<1.0 μmol/L); 10th–90th percentiles: 0.66–1.19 μmol/L | Higher baseline serum selenium was significantly inversely associated with ESCC mortality. | ^67^ |
| Esophageal Cancer | Se (serum concentration) | Prospective nested case–cohort study (590 esophageal cancer cases, 402 gastric cardia cancer cases, 87 gastric non-cardia cancer cases, 1,062 controls; selected from 29,584 participants of the General Population Trial) | Linxian, Henan Province, China (high-risk region for ESCC and gastric cardia cancer) | Healthy adults aged 40–69 years enrolled in the General Population Trial, with blood drawn in 1985 (pre-trial) and followed for 5.25 years (1986–1991) | Mean serum selenium = 72.2 μg/L (0.92 μmol/L); quartile cutoffs: Q1 ≤59.8, Q2 59.8–70.9, Q3 70.9–82.1, Q4 ≥82.1 μg/L; median values by quartile: 52, 66, 76, 92 μg/L | Higher baseline serum selenium was significantly inversely associated with incident esophageal cancer risk (RR per 11 μg/L increase = 0.89, 95% CI: 0.83–0.95; Q4 vs. Q1 RR = 0.56, 95% CI: 0.44–0.71; P-trend < 0.0001; absolute risk difference Q1 vs. Q4 = 1.23%, P < 0.0001). | ^43^ |
| Esophageal Cancer | Se (dietary intake) | Prospective cohort study (n=47,405; 201 ESCC cases) | Golestan Province, northeastern Iran (high-risk region for ESCC) | Healthy adults aged 40–75 years enrolled in 2004–2008, followed through 2014 | Median dietary selenium intake (by quartile): Q1 = 93 μg/d, Q2 = 133 μg/d, Q3 = 162 μg/d, Q4 = 197 μg/d | A U-shaped (nonlinear) association was observed between dietary selenium intake and ESCC risk (P-nonlinear = 0.001), with no significant linear trend (Q4 vs. Q1 HR = 1.12, 95% CI: 0.64, 1.94; P-trend = 0.97). | ^26^ |
| Esophageal Cancer | Se (dietary intake) | Systematic review and dose-response meta-analysis of observational studies (20 articles; 5 studies on selenium; 4,855 cases from 1,387,482 participants) | Global | General adult populations from cohort and case-control studies | Dietary selenium intake (range across studies); dose-response analysis per 10 μg/day increase | No significant association was found between dietary selenium intake and esophageal cancer risk (highest vs. lowest category OR = 0.84, 95% CI: 0.51–1.38; dose-response OR per 10 μg/day = 0.99, 95% CI: 0.97–1.01). | ^40^ |
| Esophageal Cancer | Se (dietary intake) | Population-based case-control study (218 ESCC cases, 415 controls) | Taixing, Jiangsu, China | Residents of Taixing City (high-risk region for ESCC, incidence rate 65.2/100,000 in 2000) diagnosed with incident ESCC between June–December 2000, and healthy controls randomly selected from the general population | Median dietary selenium intake among controls = 25.9 μg/d; Quartiles: Q1 (<17.0 μg/d), Q2 (17.0–25.9 μg/d), Q3 (25.9–39.3 μg/d), Q4 (≥39.3 μg/d) | Higher dietary selenium intake was significantly inversely associated with ESCC risk (Q4 vs. Q1 adjusted OR = 0.31, 95% CI: 0.13–0.70; Ptrend = 0.01). | ^40^ |
| Esophageal Cancer | Se (dietary intake) | Population-based case-control study (218 ESCC cases, 415 controls) | Taixing, Jiangsu, China | Residents of Taixing (high-risk region, incidence 65.2/100,000 in 2000) with incident ESCC diagnosed June–December 2000, and healthy controls randomly selected from the general population | Median dietary selenium intake among controls = 25.9 μg/d; quintile cutoffs: Q1 <14.6, Q2 14.6–22.1, Q3 22.1–28.9, Q4 28.9–43.2, Q5 ≥43.2 μg/d | Higher dietary selenium intake was significantly inversely associated with ESCC risk (Q5 vs. Q1 adjusted OR = 0.48, 95% CI: 0.25–0.89; P-trend = 0.002). | ^13^ |
| Esophageal Cancer | Mn (dietary intake) | Prospective cohort study (n=47,405; 201 ESCC cases) | Golestan Province, northeastern Iran (high-risk region for ESCC) | Healthy adults aged 40–75 years enrolled in 2004–2008, followed through 2014 | Dietary manganese intake (median, IQR) by quartile: Q1 6 (1.4–7) mg/d; Q2 8 (7.1–9) mg/d; Q3 10 (9.1–10.8) mg/d; Q4 12 (10.9–32.6) mg/d. | No significant association was found between dietary manganese intake and ESCC risk. | ^26^ |
| Esophageal Cancer | Mn (whole blood concentration) | Case-control study (150 cases, 177 controls) | Chaoshan region, southeastern China | ESCC pathologically confirmed at Cancer Hospital of Shantou University Medical College (2001–2020) | whole blood, median [IQR], μg/L): Cases = 18.26 (13.23–24.88); Controls = 18.17 (12.99–25.07) | Mn showed a U-shaped association with ESCC risk | ^52^ |
| Pancreatic Cancer | Zn (serum concentration) | Retrospective cohort study (n=272 patients with unresectable pancreatic cancer who started chemotherapy between April 2016 and March 2024) | Japan | Patients with unresectable pancreatic cancer whose serum zinc levels were measured before starting chemotherapy; zinc deficiency (ZD) defined as serum zinc <60 μg/dL | Serum zinc threshold for deficiency: <60 μg/dL | Zn deficiency independently predicted poorer OS. Lower serum Zn (<60 µg/dL) associated with significantly worse prognosis. | ^56^ |
| Pancreatic Cancer | Zn (dietary intake) | Systematic review and meta-analysis of observational studies (1,659 pancreatic cancer cases; total participants) | Global (included studies from the United States, Europe [Italy, UK], Australia, and Japan) | General adult populations from prospective cohort and case-control studies; age ranges varied by study (e.g., 21–92 years) | highest versus lowest categories of dietary zinc intake | Highest category (approximately >14–15 mg/day on average across studies) of dietary zinc intake was significantly associated with reduced pancreatic cancer risk | ^35^ |
| Pancreatic Cancer | Cu (serum concentration) | Retrospective case-control study (100 pancreatic cancer patients, 100 age-matched healthy controls) | Poland | Cases: advanced pancreatic cancer (Szczecin, Feb 2011–Oct 2014). Controls: matched by age, sex, smoking, and family cancer history. | Serum levels (mean [range]) were 1,432 (764–2,901) μg/L in cases and 1,098 (695–1,683) μg/L in controls, with a threshold of 1,214.58 μg/L above which pancreatic cancer risk increased. | High serum copper was significantly associated with increased pancreatic cancer risk | ^34^ |
| Pancreatic Cancer | Fe (dietary intake) | Prospective cohort study (EPIC; n=477,202 participants; 865 incident exocrine pancreatic cancer cases) | Europe (10 European countries) | Men and women aged 25–70 years recruited between 1992 and 2000 | Baseline dietary intake from uncalibrated FFQ (median [IQR], mg/day) was 14.3 [11.6–17.5] for men and 11.9 [9.6–14.4] for women, with quintile cutoffs of <12.0, 12.0–13.5, 13.5–14.9, 14.9–16.7, >16.7 for men and <10.1, 10.1–11.2, 11.2–12.2, 12.2–13.6, >13.6 for women. | Overall, dietary total iron was not significantly associated with pancreatic cancer risk. | ^46^ |
| Pancreatic Cancer | Se (serum concentration) | Retrospective case-control study (100 pancreatic cancer patients, 100 age-matched healthy controls) | Poland | Cases: advanced pancreatic cancer (Szczecin, Feb 2011–Oct 2014). Controls: matched by age, sex, smoking, and family cancer history. | Baseline levels (mean [range]) were 60 (29–122) μg/L in cases vs. 76 (43–110) μg/L in controls, and risk increased below the threshold of 67.45 μg/L. | Low serum selenium was significantly associated with increased pancreatic cancer risk | ^34^ |
| Pancreatic Cancer | Se (serum concentration) | Prospective nested case-control study within the Prostate, Lung, Colorectal, and Ovarian (PLCO) Cancer Screening Trial (303 incident pancreatic cancer cases, 606 matched controls; median follow-up 7 years, up to 16 years) | United States (10 centers nationwide) | Men and women aged 55–74 years at baseline (1993–2001); cases diagnosed through 2009; controls matched on age, sex, race, and date of blood draw | Serum selenium means were 139.0 (cases) and 142.5 (controls) ng/mL; quintile cutoffs (controls): ≤125, 125–137.6, 137.6–146.6, 146.6–160, >160; 96% had >90 ng/mL. | Overall, serum selenium was not significantly associated with pancreatic cancer risk | ^15^ |
| Pancreatic Cancer | Mn (serum and tissue concentrations) | Retrospective cohort study (138 PDAC patients, 88 healthy controls; 83 tissue pairs) | Australia | Patients with surgically resected PDAC (2004–2018) | Serum levels (mean±SD, nM) were 22.46±14.82 vs 20.82±11.70 (P=0.694) and tissue levels (μg/g) 1.242±1.234 vs 2.929±2.262 (P<0.0001), showing significance only for tissue. | Serum Mn was not different between groups, but tumor Mn decreased, and higher serum Mn predicted worse survival. | ^11^ |
| Breast Cancer | Zn (dietary intake) | Case-control study (1,591 breast cancer cases, 1,622 age-frequency matched controls) | Guangzhou, Guangdong Province, China | Female cases from three teaching hospitals (2007–2019) | Median dietary Zn (mg/day) was lower in cases for total | Total dietary Zn showed a U‑shaped association with breast cancer risk (lowest at ~10.63 mg/day) | ^63^ |
| Breast Cancer | Zn (serum concentration) | Prospective multicenter cohort study (n=1,998; 310 deaths during ~9 years of follow-up) | Sweden (SCAN-B) | Patients with newly diagnosed primary invasive breast cancer without distant metastases, enrolled between September 2010 and March 2015 | Baseline serum level (mean±SD) was 873.7±163.2 μg/L, with quartile cutoffs at ≤783, 783–862, 862–959, and >959 μg/L. | Higher zinc was linked to better survival in crude analysis but not after adjustment | ^10^ |
| Breast Cancer | Zn (serum concentration) | Prospective cohort study (n=538; 166 deaths during median ~10 years of follow-up) | Poland (Szczecin, Pomeranian Medical University) | Women diagnosed with breast cancer between 2008–2015 | Baseline serum level (mean±SD) was 847.3±129.2 μg/L, with quartile cutoffs at ≤762.7, 762.8–839.7, 840.2–919.6, and ≥920.3 μg/L. | Low serum zinc independently predicted worse 10‑year overall and breast cancer‑specific survival. | ^61^ |
| Breast Cancer | Cu (serum concentration) | Prospective cohort study (n=538; 166 deaths during median ~10 years of follow-up) | Poland (Szczecin, Pomeranian Medical University) | Women diagnosed with breast cancer between 2008–2015 | Baseline serum level (mean±SD) was 1,152.0±217.6 μg/L, with quartile cutoffs at ≤1,012.4, 1,012.7–1,122.3, 1,122.8–1,254.6, and ≥1,255.3 μg/L. | High serum copper showed a trend toward worse survival in univariate analysis but lost significance after multivariate adjustment | ^61^ |
| Breast Cancer | Cu (serum concentration) | Prospective multicenter cohort study (n=1,998; 310 deaths during ~9 years of follow-up; 13,312 person-years) | Sweden | Patients with newly diagnosed primary invasive breast cancer without distant metastases, enrolled between September 2010 and March 2015 | 1,273.6±277.6 μg/L, with quartile cutoffs at ≤1,089, 1,089–1,237, 1,237–1,415, and >1,415 μg/L. | Higher serum copper was linked to poorer survival in crude analysis, but not after multivariable adjustment. | ^10^ |
| Breast Cancer | Fe (plasma concentration) | Retrospective single-center cohort study (n=1,113; median follow-up 7 years; 111 metastatic events, 10%) | Belgium (University Hospital Leuven) | Female patients with stage I–III unilateral, unifocal invasive breast cancer diagnosed between 2007–2017 | Baseline levels (plasma, median [IQR]): 82.8 µg/dL (IQR: 60.0–100.0) | Each 10 µg/dL increment in plasma iron at diagnosis was associated with a 6% increase in metastatic risk | ^20^ |
| Breast Cancer | Zn (serum concentration) | Case-control study (73 malignant, 73 benign, 95 healthy controls) | Beijing, China | Female patients with histopathologically diagnosed benign or malignant breast tumors | Median (IQR) serum Zn (μg/dL) was 70.01 (61.89–79.57) in malignant, 73.73 (63.55–85.13) in benign, and 72.84 (67.18–81.25) in healthy controls. | Serum Zn was significantly lower in malignant breast cancer patients compared to healthy controls | ^14^ |
| Breast Cancer | Se (dietary intake) | In a cohort of 17,035 women (1,427 breast cancers, 344,584 person‑years), a nested case‑control subset (1,186 pairs) was used for serum assays. | Malmö, Sweden (low-selenium area) | Women born 1923–1950 from the Malmö Diet and Cancer Study (enrolled 1991–1996) underwent baseline dietary and serum assessments and were followed until December 2013. | Baseline dietary intake (median by quartile, μg/day) was 24.7, 30.8, 39.0, and 72.8 for Q1–Q4, respectively, with an overall mean±SD of 29±9 μg/day. | Dietary selenium showed no overall association with breast cancer risk, but a U‑shaped pattern emerged in current smokers, and serum‑dietary concordance was poor | ^9^ |
| Breast Cancer | Se (serum concentration) | Multicenter prospective cohort (n=1,996; 310 deaths, 167 recurrences) | Prospective multicenter cohort study | Patients with newly diagnosed primary invasive breast cancer without distant metastases, enrolled between 2010–2013; blood sampled at diagnosis before surgery; median age ~64 years | Baseline serum Se (median [IQR]) was 70.4 (60.1–81.3) μg/L, with Q1 cutoff <57 and Q5 cutoff >84 μg/L. | Compared with patients in the lowest quintile of serum selenium concentration (Q1), those in the highest quintile (Q5) had a 58% lower risk of all-cause mortality. | ^21^ |
| Breast Cancer | Se (serum concentration) | Prospective cohort study (n=538; mean follow-up 7.9 years; 121 deaths, 22.5%) | Szczecin, Poland | Women diagnosed with first primary invasive breast cancer between 2008–2015 | Baseline serum levels (mean [range]) were 86.2 (52.12–171.55) μg/L overall, with quartile ranges of 52.1–76.7 (Q1), 76.8–85.1 (Q2), 85.2–94.6 (Q3), and 94.7–171.5 (Q4) μg/L. | Low serum selenium predicted worse overall survival. | ^60^ |
| Breast Cancer | Se (serum concentration) | Matched case-control study (62 newly diagnosed breast cancer cases, 62 controls) | Malaysia (hospital-based) | Newly diagnosed breast cancer patients from a Malaysian hospital | Cases = 16.24 ± 8.21; Controls = 23.85 ± 9.80; Mean difference was statistically significant (t = 5.09, P < 0.001) | Serum selenium was significantly inversely associated with breast cancer risk: each 1 μg/dL increase in serum selenium reduced breast cancer risk by 11% | ^54^ |
| Breast Cancer | Se (dietary intake) | Case-control study (1,591 breast cancer cases, 1,622 age-frequency matched controls) | Guangzhou, Guangdong Province, China | Female cases from three teaching hospitals (2007–2019) | Median energy‑adjusted dietary Se (μg/day) was lower in cases than controls for total (48.00 vs 49.12, P=0.004) | Total dietary Se was not significantly associated with breast cancer risk | ^11^ |
| Breast Cancer | Mn (serum and hair concentrations) | Meta-analysis | Systematic review and meta-analysis of case-control studies (11 eligible studies; 1,302 subjects) | Breast cancer patients and healthy controls from case-control studies; all studies were from Asian populations | Not applicable | Mn was significantly lower in breast cancer patients versus controls, and the reduction was greater in hair than serum. | ^57^ |
| Prostate Cancer | Zn (serum concentration) | Case-control study (79 prostate cancer patients, 74 male healthy controls) | Southwest China (West China Hospital, Sichuan University) | Men diagnosed with prostate cancer by biopsy (April 2021–June 2022) | Baseline serum levels (median [IQR]) were 657 (524–846) μg/L in cases versus 820 (665–993) μg/L in controls (P < 0.001). | Serum Zn was significantly lower in prostate cancer patients than controls, was identified as an independent protective factor | ^72^ |
| Prostate Cancer | Zn (serum concentration) | Case-control study (40 PCa patients, 22 BPH patients, 30 healthy male controls) | Saudi Arabia | Newly diagnosed prostate cancer patients; BPH patients; healthy male controls with similar socio-economic status | Serum levels (mean±SD, μg/mL) were 0.51±0.09 in PCa vs 0.82±0.19 in total controls (P<0.05). | Serum Zn was significantly lower in prostate cancer patients compared to BPH and healthy controls | ^55^ |
| Prostate Cancer | Zn (dietary intake) | Population-based case-control study (733 incident prostate cancer cases, 1,228 population-based controls) | Spain (MCC-Spain) | Men aged 20–85 years with incident histologically-confirmed prostate cancer | Baseline dietary Zn (mean±SD) was 9.91±2.98 in cases vs 9.68±2.94 in controls (P=0.096) | Higher dietary Zn showed a borderline positive association with prostate cancer risk, stronger in low‑grade and localized tumors | ^25^ |
| Prostate Cancer | Zn (dietary intake) | Prospective population-based cohort study (n=525; 218 prostate cancer deaths, 257 other deaths) | Örebro, Sweden | Men aged <80 years with newly diagnosed prostate cancer between 1989–1994 | Baseline dietary Zn (mean±SD) was 14.2±4.1 mg/day overall, with energy‑adjusted quartile ranges of 9.0–12.8 (Q1), 12.8–14.1 (Q2), 14.1–15.6 (Q3), and 15.6–20.1 (Q4) mg/day. | Higher dietary zinc intake was significantly associated with reduced prostate cancer-specific mortality | ^23^ |
| Prostate Cancer | Zn (dietary intake) | A case‑control study (127 cases, 81 controls) and a meta‑analysis (17 studies, 111,199 participants, 11,689 cases) were conducted. | Multiple | A hospital‑based case‑control study (African American men aged 40–85 with newly diagnosed prostate cancer vs healthy controls without family history) and a meta‑analysis of 17 general‑population studies (1988–2013) | Mean dietary Zn (mg/day) was 10.8±7.4 in cases vs 14.3±15.4 in controls (P=0.06), with tertile cutoffs at <6.5, 6.5–12.5, and >12.5. | Overall zinc intake was not significantly associated with prostate cancer risk, though case‑control studies showed a significant 21% increase while cohort and biomarker studies did not | ^41^ |
| Prostate Cancer | Cu (serum concentration) | Case-control study (79 prostate cancer patients, 74 male healthy controls) | Southwest China (West China Hospital, Sichuan University) | Men diagnosed with prostate cancer by biopsy (April 2021–June 2022) | Serum Cu (median [IQR]) was 645 (560–730) μg/L in cases vs 730 (630–850) in controls | Serum Cu was significantly lower in prostate cancer patients than controls and emerged as an independent protective factor | ^72^ |
| Prostate Cancer | Cu (serum concentration) | Case-control study (40 PCa patients, 22 BPH patients, 30 healthy male controls) | Saudi Arabia | Newly diagnosed prostate cancer patients, BPH patients, and healthy male controls | Baseline serum (mean±SD) was 1.69±0.31 μg/mL in PCa vs 1.02±0.24 in total controls (P<0.005) | Serum Cu was significantly higher in prostate cancer patients compared to BPH and healthy controls | ^55^ |
| Prostate Cancer | Cu (serum concentration) | Systematic review and meta-analysis (11 studies; 622 prostate cancer patients, 564 controls; literature search up to December 2024) | Global | Prostate cancer patients and healthy controls from case-control studies; age ranges varied across individual studies | Not applicable | Serum copper levels were significantly higher in prostate cancer patients compared to controls | ^22^ |
| Prostate Cancer | Fe (serum concentration) | Case-control study (40 PCa patients, 22 BPH patients, 30 healthy male controls) | Saudi Arabia | Newly diagnosed prostate cancer patients, BPH patients, and healthy male controls | Serum levels (mean±SD, μg/mL) were 1.96±0.58 in PCa vs 1.24±0.22 in total controls (P<0.05). | Serum Fe was significantly higher in prostate cancer patients compared to BPH and healthy controls | ^55^ |
| Prostate Cancer | Ferritin (Ferritin, serum iron, and transferrin concentration) | Prospective case‑cohort study (random subcohort n = 2,738; prostate cancer cases n = 554) | Heidelberg, Germany (EPIC‑Heidelberg) | Male participants aged 35–65 years from the local general population. | Baseline levels: Prostate cancer cases: ferritin 288.8 ± 235.0 μg/L; iron 18.2 ± 6.0 μmol/L; transferrin 31.9 ± 4.5 μmol/L; Subcohort men: ferritin 287.4 ± 221.0 μg/L; iron 18.1 ± 6.0 μmol/L; transferrin 32.0 ± 4.3 μmol/L. | No significant associations were observed between any iron status marker and prostate cancer risk. | ^53^ |
| Prostate Cancer | Fe (serum concentration) | Systematic review and meta-analysis (6 studies; 318 prostate cancer patients, 315 controls; literature search up to December 2024) | Global | Prostate cancer patients and healthy controls from case-control studies | Not applicable | Overall, serum iron did not significantly differ between prostate cancer cases and controls | ^22^ |
| Prostate Cancer | Se (serum concentration) | Case-control study (79 prostate cancer patients, 74 male healthy controls) | Southwest China (West China Hospital, Sichuan University) | Men diagnosed with prostate cancer by biopsy (April 2021–June 2022) | Baseline serum (median [IQR]) was 91.0 (77.6–102.0) μg/L in cases vs 106.0 (88.8–123.0) in controls | Serum Se was significantly lower in prostate cancer patients than controls, and was independently protective effect with prostate cancer incidence | ^72^ |
| Prostate Cancer | Se (serum concentration) | Nested case-control within prospective cohort (EPIC; n=959 cases / 1,059 controls) | 10 European countries | Men (43–76 years, cancer‑free at baseline) with incident prostate cancer detected via registries/follow‑up; controls matched for center, age, blood draw time, and fasting. | Baseline plasma Se (geometric mean) was 70.6 vs 71.9 μg/L in cases vs controls (P=0.49), with quintile cutoffs (controls) at ≤62, 62–68.5, 68.5–75, 75–84, and ≥84.1 μg/L. | Overall, plasma selenium was not significantly associated with prostate cancer risk | ^3^ |
| Prostate Cancer | Se (serum/plasma concentration) | Systematic review and meta-analysis (17 studies: 12 case-control, 4 cohort, 1 RCT; 6,136 prostate cancer cases, >34,901 participants; literature search up to September 2016) | Multiple | General adult male populations from cohort, case-control, and randomized controlled trials; age ranges varied across studies | Not applicable | Higher serum selenium levels were significantly associated with reduced prostate cancer risk | ^18^ |
| Prostate Cancer | Se (plasma concentration) | Nested case-control study within the Physicians' Health Study (PHS) (1,286 prostate cancer cases, 1,267 controls) | United States (Physicians' Health Study) | Male US physicians (aged 40–84, Caucasian, enrolled 1982) | Baseline plasma selenium (median [10th–90th percentile]) was 0.109 (0.086–0.133) μg/g in cases vs 0.110 (0.088–0.134) in controls. | Among prostate cancer patients carrying the G allele (GG or AG genotype) of the SEP15 gene rs561104 polymorphism, higher plasma selenium levels were significantly associated with reduced prostate cancer-specific mortality | ^49^ |
| Prostate Cancer | Se (plasma concentration) | Systematic review and meta-analysis (8 studies; 711 prostate cancer patients, 907 controls; literature search up to December 2024) | Global | Prostate cancer patients and healthy controls from case-control studies | Not applicable | Serum selenium levels were significantly lower in prostate cancer patients compared to controls | ^22^ |
| Prostate Cancer | Mn (Serum concentration) | Case-control study (40 PCa patients, 22 BPH patients, 30 healthy male controls) | Saudi Arabia | Newly diagnosed prostate cancer patients; BPH patients; healthy male controls with similar socio-economic status | Serum levels (mean±SD, μg/mL) were 0.001±0.001 in PCa vs 0.0024±0.001 in total controls | Serum Mn was significantly lower in prostate cancer patients compared to BPH and healthy controls | ^55^ |
| Prostate Cancer | Mn (serum concentration) | Systematic review and meta-analysis (4 studies; 253 prostate cancer patients, 216 controls; literature search up to December 2024) | Global | Prostate cancer patients and healthy controls from case-control studies | Not applicable | Serum manganese levels were lower in prostate cancer patients compared to controls, but the difference was not statistically significant | ^22^ |
| Lung Cancer | Zn (plasma concentration) | Nested case-control study (101 incident lung cancer cases, 202 healthy controls) | China (Shiyan City, Hubei Province) | Retired workers from Dongfeng Motor Corporation (DMC) enrolled in the DFTJ cohort at baseline (2008–2010) | Not reported | sphingomyelin mediates 41.7% of zinc-associated lung cancer protection and independently predicts lower risk | ^6^ |
| Lung Cancer | Zn (serum concentration) | Systematic review and meta-analysis of observational studies (32 studies: 31 case-control, 1 observational; 2,894 lung cancer cases, 9,419 controls; literature search up to December 2018) | Global | Lung cancer patients and healthy controls from case-control and observational studies; age ranges varied across individual studies | Not applicable | Serum zinc levels were significantly lower in lung cancer patients compared to controls | ^66^ |
| Lung Cancer | Zn (serum concentration) | Hospital-based case-control study (146 lung cancer patients, 146 healthy controls) | Northeast China (Changchun, Jilin Province; Han Chinese population) | Newly diagnosed primary lung cancer patients from the Cancer Center of The First Hospital of Jilin University (2011–2015) | Baseline serum (median [IQR]) was 1.02 (0.50) mg/L in cases vs 0.93 (0.32) in controls | Higher serum Zn was associated with increased lung cancer risk | ^65^ |
| Lung Cancer | Cu (serum concentration) | Hospital-based case-control study (146 lung cancer patients, 146 healthy controls) | Northeast China (Changchun, Jilin Province; Han Chinese population) | Newly diagnosed primary lung cancer patients from the Cancer Center of The First Hospital of Jilin University (2011–2015) | Baseline serum (median [IQR]) was 1.76 (0.80) mg/L in cases vs 1.04 (0.33) in controls | Higher serum Cu was significantly associated with increased lung cancer risk | ^65^ |
| Lung Cancer | Cu (serum concentration) | Hospital-based case-control study (154 NSCLC patients, 154 controls) | China (Anhui Province) | Patients with primary non-small cell lung cancer from the First Affiliated Hospital of Anhui Medical University (2006–2007) | Baseline levels (serum, mean ± SD, ng/mL): Cases = 1,623.6 ± 818.3; Controls = 1,285.1 ± 523.8 (P < 0.01) | Higher serum Cu (>1,500 ng/mL) was significantly associated with increased NSCLC risk | ^30^ |
| Lung Cancer | Fe (Ferritin concentration) | Systematic review and meta-analysis (5 studies; 20 articles total in the review) | Global | Lung cancer patients and healthy controls from case-control and cohort studies | Not applicable | Serum ferritin concentrations were significantly higher in lung cancer patients than in healthy controls | ^64^ |
| Lung Cancer | Fe (serum concentration) | Matched case-control study (200 lung cancer patients, 200 healthy controls) | Poland (Szczecin, Pomeranian Medical University) | Consecutive lung cancer patients diagnosed at the Clinical Thoracic Surgery Department and International Hereditary Cancer Centre | Serum mean was 1,399.73 μg/L in cases vs 1,194.66 in controls; quartile cutoffs: <942.90, 942.90–1,182.65, 1,182.65–1,497.00, >1,497.00. | Higher serum iron was significantly associated with increased lung cancer risk | ^59^ |
| Lung Cancer | Se (serum/plasma concentration and supplementation) | Systematic review and meta-analysis of 5 randomized controlled trials, 26 observational studies | Global | General adult populations, lung cancer patients, and high-risk populations from RCTs and observational studies | Serum selenium levels varied widely across studies. In the Nutritional Prevention of Cancer (NPC) trial, tertile cutoffs were <106, 106–121.6, and ≥121.6 ng/mL; participants in the Selenium and Vitamin E Cancer Prevention Trial (SELECT) had a median of ~135 ng/mL (reflecting a selenium-replete U.S. population), while low-selenium populations (e.g., China) averaged ~70 ng/mL. Protective effects were observed primarily in those with baseline selenium <106 ng/mL | Selenium supplementation may reduce lung cancer risk at low baseline Se (<106 ng/mL) but increase risk at high levels (≥121.6 ng/mL), suggesting a U‑shaped or biphasic dose–response. | ^24^ |
| Lung Cancer | Se (whole blood, plasma, and lung tissue concentrations) | Case-control study (84 lung cancer patients, 61 healthy controls; tumor and adjacent normal lung tissue from 57 patients at surgery) | Poland | Lung cancer patients undergoing surgery; healthy controls from the same region; blood samples collected before treatment; tissue samples obtained at the time of surgery | Whole blood Se: Patients = 61.6 ± 20.5 ng/mL; Controls = 80.3 ± 20.0 ng/mL (P < 0.001) — 23% lower  Plasma Se: Patients = 43.5 ± 14.8 ng/mL; Controls = 56.2 ± 14.0 ng/mL (P < 0.001) — 23% lower  Tumor tissue Se: 141.8 ± 44.3 ng/g wet weight (vs. adjacent normal 85.1 ± 33.6; P < 0.0001) — 67% higher | Blood Se was significantly lower in lung cancer patients than controls, while tumor tissue Se was significantly elevated compared to adjacent normal lung tissue | ^71^ |
| Colorectal Cancer | Zn (serum concentration) | Prospective cohort (n=116; stages I–III colon cancer) | China (Nanjing, Nantong) | Patients with stages I–III colon cancer admitted to two hospitals (March 2016–June 2017) | Baseline levels (serum, mean ± SD): Overall = 856.0 ± 322.5 µg/L. Patients were divided into Low Zn (<856.0 µg/L; n=53) and High Zn (≥856.0 µg/L; n=63) groups. | Low serum Zn was significantly associated with higher distant metastasis incidence and lower 2-year survival | ^68^ |
| Colorectal Cancer | Cu (serum/plasma/blood concentration) | Systematic review and meta-analysis (26 studies; 2,578 colorectal cancer cases, 7,050 controls; 9,628 total participants) | Global | Colorectal cancer patients and healthy controls from case-control studies | Not applicable | Overall, CRC patients had higher mean Cu levels than controls, but the difference was not statistically significant | ^58^ |
| Colorectal Cancer | Fe (dietary intake) | Prospective cohort study (EPIC; 450,105 participants; 6,162 incident CRC cases) | 10 European countries | Men and women aged 35–75 years recruited between 1992–2000 | Baseline levels (dietary intake, mean ± SD, mg/day):  Men: Cases = 14.5 ± 4.7; Non-cases = 14.6 ± 4.7  Women: Cases = 11.8 ± 3.5; Non-cases = 12.2 ± 3.8 | Total iron intake was not significantly associated with CRC risk in men (HR Q5 vs. Q1 = 0.88, 95% CI: 0.73–1.06) or women (HR Q5 vs. Q1 = 1.11, 95% CI: 0.94–1.31). | ^2^ |
| Colorectal Cancer | Fe (Ferritin serum concentration) | Retrospective cohort study (120 relapsed or refractory mCRC patients) | Korea | Cytologically confirmed mCRC patients aged ≥20 years who had relapsed or refractory disease from standard chemotherapy or were unqualified for conventional treatment | Baseline ferritin: high (≥150 ng/mL) vs low (<150 ng/mL) | Patients with high serum ferritin (≥150 ng/mL) had significantly poorer overall survival | ^33^ |
| Colorectal Cancer | Se (prediagnostic serum concentration) | Nested case-control within prospective cohort (EPIC; n=995 CRC cases) | Europe | CRC cases from the EPIC cohort with available serum Se | Se: Overall not provided in abstract; quintile cutoffs: Q1 ≤67.5, Q2 67.6–77.4, Q3 77.5–88.0, Q4 88.1–99.9, Q5 ≥100.0; range: 29–142 µg/L | Higher prediagnostic serum Se was associated with lower CRC-specific mortality and lower overall mortality | ^7^ |
| Colorectal Cancer | Se (serum concentration) | Nested case-control study within the Women's Health Initiative (WHI) Observational Study (804 colorectal cancer cases, 805 matched controls; follow-up from 1993–2005) | United States (40 clinical centers nationwide) | Postmenopausal women aged 50–79 years at recruitment | Serum Se (mean±SD, μg/L) was 137.8±24.3 in cases vs 135.6±21.1 in controls (P=0.05), range 81.0–398.7, with quintile cutoffs ≤117.5, 117.6–128.5, 128.5–138.7, 138.7–152.6, and >152.6 | Serum selenium was not significantly associated with colorectal cancer risk in women | ^62^ |
| Colorectal Cancer | Se (serum concentration) | Case-control study (201 colorectal cancer patients, 15 age-matched healthy controls; from the Moravian region of the Czech Republic) | Czech Republic (Moravian region) | Patients with colorectal adenocarcinoma from the Teaching Hospital Olomouc; age-matched healthy controls from the same region | Baseline levels (serum, mean ± SD): Cases = 41.8 ± 11.6 µg/L; Controls = 76.6 ± 10.8 µg/L | Serum Se was significantly lower in colorectal cancer patients than in healthy controls | ^45^ |
| Colorectal Cancer | Se (dietary intake) | Case-control (n=683 cases / 759 controls) | Lesser Poland, Central Europe | Incident CRC cases (sporadic, age ≥40 years) and hospital-based controls from University Hospital in Krakow and Narutowicz Municipal Hospital | Selenium: Cases = 49 μg/day (39–59); Controls = 50 μg/day (38–64); Mean ± SD: Cases = 51 ± 17; Controls = 55 ± 27 μg/day | Dietary selenium was significantly inversely associated with CRC risk: each 10 μg/day increase in selenium was associated with an 8% reduction in CRC odds | ^5^ |
| Colorectal Cancer | Se (tissue concentration) | Systematic review and meta-analysis (6 studies on tissue specimens; 104 non-cancerous tissue controls, 115 CRC malignant tissue specimens) | Global (4 European, 2 North American studies) | CRC patients with paired or unpaired cancerous and adjacent normal colon tissue specimens | Not applicable | CRC malignant tissue had significantly higher Se levels than matched non-cancerous colon tissue | ^47^ |
| Colorectal Cancer | Mn (serum concentration) | Case-control study (201 CRC patients, 15 age-matched healthy controls) | Czech Republic (Moravian region) | Patients with colorectal adenocarcinoma from the Teaching Hospital Olomouc | Baseline levels (serum, mean ± SD): Cases = 16.3 ± 4.5 µg/L; Controls = 10.4 ± 1.7 µg/L (P < 0.001) | Serum Mn was significantly higher in colorectal cancer patients than in healthy controls | ^45^ |
| Ovarian Cancer | Zn (serum/plasma concentration) | Systematic review and meta-analysis of case-control studies (20 studies; 699 ovarian cancer patients, 567 benign ovarian lesion patients, 1,194 healthy controls) | Global (multiple countries; primarily Asian populations) | Women with ovarian cancer, benign ovarian lesions, and healthy controls from case-control studies; age and demographic characteristics varied across individual studies | Not applicable | Ovarian cancer patients had significantly lower circulating zinc concentrations than healthy controls (pooled SMD = -1.01, 95% CI: -1.38 to -0.64) and than patients with benign ovarian lesions (SMD = -0.55, 95% CI: -0.81 to -0.28). | ^36^ |
| Ovarian Cancer | Cu (serum/plasma concentration) | Systematic review and meta-analysis of case-control studies (20 studies; 699 ovarian cancer patients, 567 benign ovarian lesion patients, 1,194 healthy controls) | Global (multiple countries; primarily Asian populations) | Women with ovarian cancer, benign ovarian lesions, and healthy controls from case-control studies; age and demographic characteristics varied across individual studies | Not applicable | Ovarian cancer patients had significantly higher circulating copper concentrations than healthy controls (pooled SMD = 1.46, 95% CI: 0.82 to 2.09) and than patients with benign ovarian lesions | ^36^ |
| Ovarian Cancer | Fe (Ferritin, serum concentration) | Case-control study (60 ovarian cancer patients, 60 controls) | Italy (University Medical School of Bari) | Patients with primary epithelial ovarian carcinoma surgically treated between March 1993 and September 1996 | Baseline levels (serum ferritin, mean ± SD, ng/mL): Ovarian cancer patients = 112.3 ± 21.2 (range: 9–947); Healthy controls = 54.7 ± 7.8 (range: 14–135) | Serum ferritin was significantly higher in ovarian cancer patients than healthy controls | ^50^ |
| Ovarian Cancer | Fe (serum concentration) | Case-control study (60 ovarian cancer patients, 60 controls) | Italy (University Medical School of Bari) | Patients with primary epithelial ovarian carcinoma surgically treated between March 1993 and September 1996 | Baseline levels (serum iron, mean ± SD, μg/dL): Ovarian cancer patients = 57.2 ± 3.8 (range: 18–119); Healthy controls = 66.3 ± 2.61 (range: 21–122) | No significant difference in serum iron levels was observed between ovarian cancer patients and healthy controls | ^50^ |
| Ovarian Cancer | Se (serum concentration) | Case-control study (157 ovarian cancer patients, 157 age- and BMI-matched controls) | Poland | Patients with newly diagnosed ovarian cancer (confirmed by intraoperative histopathology) treated between 2015 and 2019 | Serum Se (μg/L) was markedly lower in ovarian cancer cases (mean 53.31, median 52.89) than controls (78.99, 77.72), with advanced FIGO III–IV showing the lowest levels (mean 49.86, median 48.86). | Ovarian cancer patients had significantly lower serum Se concentrations than controls | ^32^ |
| Ovarian Cancer | Mn (whole blood concentration) | Case-control study (n=72; 26 cases, 46 controls) | Ankara, Turkey | Patients with primary epithelial ovarian tumors (serous, endometrioid, mixed, clear cell) who underwent surgical resection | Median whole blood Mn: 9.01 μg/L in cases vs. 12.55 μg/L in controls. | Blood Mn levels were significantly lower in all EOC histologic subtypes compared to controls | ^12^ |
| Liver Cancer | Zn (dietary intake) | Matched case-control study (434 HCC cases, 434 sex- and age-matched controls) | China (Guangdong Province: cases from Sun Yat-sen University Cancer Center; controls from Guangzhou Nutrition and Health Study community-based cohort) | Patients with newly diagnosed HCC enrolled in the Guangdong Liver Cancer Cohort (GLCC) between 2013 and 2020 | Dietary intake (median, IQR; mg/day, energy-adjusted by residual method): Cases: Zn = 12.3 (11.1–13.6); Controls: Zn = 13.0 (12.0-14.0). | Higher dietary Zn intake was significantly associated with lower HCC risk | ^38^ |
| Liver Cancer | Zn (serum concentration) | Matched case-control study (434 HCC cases, 434 sex- and age-matched controls) | China (Guangdong Province: cases from Sun Yat-sen University Cancer Center; controls from Guangzhou Nutrition and Health Study community-based cohort) | Patients with newly diagnosed HCC enrolled in the Guangdong Liver Cancer Cohort (GLCC) between 2013 and 2020 | Serum levels (median, IQR; μg/L): Cases: Zn = 871 (773–980); Controls: Zn = 1098 (968–1257) | Higher serum Zn levels were strongly associated with reduced HCC risk | ^38^ |
| Liver Cancer | Cu (dietary intake) | Matched case-control study (434 HCC cases, 434 sex- and age-matched controls) | China (Guangdong Province: cases from Sun Yat-sen University Cancer Center; controls from Guangzhou Nutrition and Health Study community-based cohort) | Patients with newly diagnosed HCC enrolled in the Guangdong Liver Cancer Cohort (GLCC) between 2013 and 2020 | Dietary intake (median, IQR; mg/day, energy-adjusted by residual method): Cases: Cu = 1.56 (1.21–2.04); Controls: Cu = 2.10 (1.63–2.89). | Dietary Cu intake was not significantly associated with HCC risk after full multivariable adjustment | ^38^ |
| Liver Cancer | Cu (serum concentration) | Matched case-control study (434 HCC cases, 434 sex- and age-matched controls) | China (Guangdong Province: cases from Sun Yat-sen University Cancer Center; controls from Guangzhou Nutrition and Health Study community-based cohort) | Patients with newly diagnosed HCC enrolled in the Guangdong Liver Cancer Cohort (GLCC) between 2013 and 2020 | Serum levels (median, IQR; μg/L): Cases: Cu = 982 (838–1200); Controls: Cu = 874 (802–955). | Serum Cu levels were significantly higher in HCC cases than controls (median 982 vs. 874 μg/L) and were positively associated with HCC risk | ^38^ |
| Liver Cancer | Fe (serum concentration) | Retrospective cohort (n=18,569 NAFLD patients; 244 HCC; mean 4.34 years follow-up) | United States | Patients aged 40–89 years with a clinical diagnosis of NAFLD | Serum iron (median, 5th–95th percentile; μg/dL): HCC cases (n=188) = 70.5 (19.0–171.0); HCC-free (n=15,586) = 69.0 (19.0–141.0) | Elevated serum iron (>175 μg/dL) was significantly associated with a 2.91-fold increased risk of HCC compared with normal range (75–175 μg/dL), after adjusting. Low serum iron (<75 μg/dL) was not significantly associated with HCC risk. | ^70^ |
| Liver Cancer | Se (serum concentration) | Nested case-control within prospective cohort | 10 European countries | European Prospective Investigation into Cancer and Nutrition (EPIC) cohort participants aged 25-70 years at recruitment (1992-2000) | Geometric mean serum Se: 71.3 μg/L in cases vs. 85.2 μg/L in controls | Higher serum Se was significantly associated with lower HCC risk | ^27^ |
| Liver Cancer | Mn (dietary intake) | Prospective cohort study (n = 36,100 participants, 239 incident liver cancer cases) | Japan | Japanese residents aged 40-79 years at baseline (1988-1990) with no history of cancer; cancer follow-up data available in 24 of 45 study areas | Energy-adjusted dietary Mn intake (median by quintile): Men: Q1 = 3.0 mg/d, Q5 = 10.4 mg/d; Women: Q1 = 2.7 mg/d, Q5 = 9.3 mg/d. | Higher dietary Mn was inversely associated with liver cancer risk only in men, especially those without liver diseases, with no effect in women or in men with liver disease; dietary sources showed no associations. | ^31^ |
| Liver Cancer | Mn (serum concentration) | Systematic review and meta-analysis (6 studies; 272 HCC cases, 248 healthy controls) | Asia | HCC patients confirmed by pathology or other methods; healthy individuals as controls | Serum Mn was lower in HCC cases than controls across studies, with wide ranges (~0.67 μmol/L to 27.48 μg/L in cases; ~0.49 μmol/L to 29.35 μg/L in controls), though values varied. | Serum Mn levels in HCC patients were significantly lower than in healthy controls (pooled SMD = -0.941, 95% CI: -1.559 to -0.323). | ^17^ |
| Bladder Cancer | Zn (serum concentration) | Systematic review and meta-analysis (3 studies; total participants varied; case-control and cross-sectional designs) | Primarily Asian populations | Bladder cancer patients | Bladder cancer patients had significantly lower serum zinc levels than healthy controls across three studies (pooled SMD = −1.072, 95% CI: −1.489 to −0.656, P < 0.0001), with mean case values ranging from ~0.62 µg/mL to 96.62 µg/L versus ~0.96 µg/mL to 121.93 µg/L in controls. | Bladder cancer patients had significantly lower serum Zn levels compared with controls | ^42^ |
| Bladder Cancer | Cu (serum concentration) | Systematic review and meta-analysis (3 studies; total participants varied; case-control and cross-sectional designs) | Primarily Asian populations | Primarily Asian populations | Bladder cancer patients exhibited significantly higher serum copper levels than healthy controls across three studies (pooled SMD = 1.069, 95% CI: 0.302 to 1.836, P = 0.006), with mean case values ranging from ~0.71 µg/mL to 143.2 µmol/L versus ~0.65 µg/mL to 104.9 µmol/L in controls. | Bladder cancer patients had significantly higher serum Cu levels compared with controls | ^42^ |
| Bladder Cancer | Fe (serum concentration) | Cross-sectional case-control study (n = 109; 51 cases, 58 controls) | Isfahan, Iran | Patients with histologically confirmed bladder cancer | Serum Fe (median, interquartile range): Cases: 4.43 (2.25–6.79) μg/mL; Controls: 7.77 (5.25–9.86) μg/mL | Serum Fe levels were significantly lower in bladder cancer patients compared to controls | ^44^ |
| Bladder Cancer | Fe (Heme iron, dietary intake) | Prospective cohort study (n = 481,419 participants; 1,001 incident bladder cancer cases) | 10 European countries (Denmark, France, Germany, Greece, Italy, the Netherlands, Norway, Spain, Sweden, United Kingdom) from the EPIC study | General population aged 25-70 years at recruitment (1992-2000) | Heme iron quartiles (mg/d): Q1: <0.6; Q2: >0.6–1.02; Q3: >1.02–1.53; Q4: >1.53–4.0. | No significant association was found between dietary heme iron intake and bladder cancer risk. | ^29^ |
| Bladder Cancer | Se (serum concentration) | A meta‑analysis (7 studies, 1,910 cases, 17,339 controls) included serum Se measurements in 3 studies. | USA (4 studies) and Northern Europe (3 studies; Belgium, Finland, Netherlands) | Bladder cancer cases | High serum Se defined as ≥100 μg/L (optimal ~80–95 μg/L), though individual studies used varying cutoffs. | Higher serum Se levels were significantly associated with reduced bladder cancer risk | ^4^ |
| Bladder Cancer | Mn (blood and urine concentrations) | Case-control study (n = 200; 100 cases, 100 controls) | India | Bladder cancer cases | Blood Mn: Cases 38.62 ± 46.86 μg/L vs. Controls 11.06 ± 8.25 μg/L; reference range 4.2–16.5 μg/L. Urinary Mn: Cases 2.64 ± 4.34 μg/L vs. Controls 1.40 ± 3.60 μg/L; reference range <1.4 μg/L. | Blood and urinary Mn levels were significantly higher in bladder cancer patients compared to controls. | ^48^ |
| Kidney Cancer | Zn (serum concentration) | Case-control study (n = 172; 100 cases, 72 controls) | Shandong Province, China | Patients with first pathologically confirmed primary kidney tumor | Serum Zn median (IQR): Cases 953 (802–1074) μg/L; Controls 1418 (1030–1532) μg/L (P < 0.001). | Serum Zn levels were significantly lower in kidney tumor patients compared to controls. | ^73^ |
| Kidney Cancer | Zn (blood concentration) | Prospective cohort study (n = 284 kidney cancer patients) | Szczecin, Poland | Unselected kidney cancer patients | The blood zinc quartiles (µg/L) were: QI 0.68–5708.67, QII 5724.69–6374.45, QIII (reference) 6375.10–6943.56, and QIV 6946.63–9206.50. | Patients with lower serum Zn had higher HRs compared to those with higher Zn levels, but no statistical significance in multivariable analysis (Supplementary Table S15). | ^76^ |
| Kidney Cancer | Cu (blood concentration) | Prospective cohort study (n = 284 kidney cancer patients) | Szczecin, Poland | Unselected kidney cancer patients | Blood Cu quartiles (µg/L): QI: 2.68–845.23; QII: 845.77–928.81 (reference); QIII: 932.32–1033.68; QIV: 1038.30–1674.10. | Highest blood Cu quartile was significantly associated with increased all-cause mortality compared to the reference QII. Association was stronger in women | ^76^ |
| Kidney Cancer | Fe (serum concentration) | Case-control study (n = 172; 100 cases, 72 controls) | Shandong Province, China | Patients with first pathologically confirmed primary kidney tumor | Serum Fe median (IQR): Cases 3975 (2541–5047) μg/L; Controls 4380 (2965–5487) μg/L. | Serum Fe levels were significantly lower in kidney tumor patients compared to controls. | ^73^ |
| Kidney Cancer | Se (serum concentration) | Case-control study (n = 172; 100 cases, 72 controls) | Shandong Province, China | Patients with first pathologically confirmed primary kidney tumor | Serum Se median (IQR): Cases 84.5 (76.5–98.3) μg/L; Controls 87.3 (79.2–99.8) μg/L (P > 0.05; not significant). | No significant difference in serum Se levels was observed between kidney tumor patients and controls overall. | ^73^ |
| Kidney Cancer | Mn (serum concentration) | Case-control study (n = 172; 100 cases, 72 controls) | Shandong Province, China | Patients with first pathologically confirmed primary kidney tumor | Serum Mn median (IQR): Cases 64.1 (42.3–94.5) μg/L; Controls 95.4 (58.1–123.0) μg/L. | Serum Mn levels were significantly lower in kidney tumor patients compared to controls. | ^73^ |
| Oral Cancer | Zn (serum concentration) | Hospital‑based case‑control study (n=344 cases / 1,122 controls) | Fujian, China | Incident, histologically confirmed primary oral cancer patients | Serum Zn median (IQR): Cases 1711.0 (1052.5–2859.5) μg/L; Controls 1027.5 (840.0–1212.0) μg/L (P < 0.001). Restricted cubic spline revealed a U‑shaped relationship; optimal cutoff points: normal range 508–1381 μg/L; low <508 μg/L; high ≥1382 μg/L. | Both deficient and elevated serum Zn levels were significantly associated with oral cancer risk. | ^16^ |
| Oral Cancer | Cu (serum concentration) | Hospital‑based case‑control study (n=344 cases / 1,122 controls) | Fujian, China | Incident, histologically confirmed primary oral cancer patients | Serum Cu median (IQR): Cases 958.5 (716.0–1223.0) μg/L; Controls 995.5 (836.0–1237.0) μg/L (P = 0.014). Restricted cubic spline revealed a U‑shaped relationship; optimal cutoff points: normal range 697–1648 μg/L; low <697 μg/L; high ≥1649 μg/L. | Both deficient and elevated serum Cu levels were significantly associated with oral cancer risk. | ^16^ |
| Oral Cancer | Se (serum concentration) | Hospital‑based case‑control study (n = 641; 235 cases, 406 controls; enrollment September 2011 – December 2018) | Fujian Province, China | Incident, histologically confirmed primary oral cancer patients | Serum Se median (IQR): Cases 115.25 (78.29–148.48) μg/L; Controls 154.39 (140.30–175.94) μg/L (P < 0.001). Tertile cut‑points based on control distribution: T1 (lowest), T2, T3 (highest). | Higher serum Se levels were significantly associated with lower oral cancer risk. | ^8^ |
| Oral Cancer | Fe (serum concentration) | Case-control study (n = 100; 50 oral cancer patients, 50 healthy controls) | Mangalore, India | Oral cancer patients from the Department of Oral Surgery vs. healthy controls with no complications. | Serum iron (mean ± SD): Cases 68.00 ± 34.25 μg/dL; Controls 110 ± 17.00 μg/dL. | Serum iron levels were significantly reduced in oral cancer patients compared to healthy controls | ^39^ |
| Oral Cancer | Mn (serum concentration) | Case-control study (n = 1,887; 468 cases, 1,419 controls) | Fujian Province, China | Newly diagnosed oral cancer patients from the Department of Oral and Maxillofacial Surgery | Case median serum Mn was 0.001 vs 2.381 μg/L in controls, but many case values likely below detection limit. | Higher serum Mn levels were significantly associated with a lower risk of oral cancer | ^37^ |
| Cervical Cancer | Zn (serum concentration) | Systematic review and meta-analysis (12 case-control studies; total: 591 cervical cancer cases, 946 controls) | Asia | Patients with cervical cancer vs. controls without cervical cancer from case-control studies. | Serum zinc levels varied widely across studies with different units; among μmol/L studies, case means ranged 5.74–23.51 and control means 7.25–133.9, with two control means >100 μmol/L likely driving substantial heterogeneity. | Serum zinc levels were significantly lower in cervical cancer patients compared to controls | ^69^ |
| Cervical Cancer | Cu (serum concentration) | Systematic review and meta‑analysis (14 case‑control studies; total: 747 cervical cancer cases, 1,014 controls) | Asia | Patients with cervical cancer vs. controls without cervical cancer from case‑control studies. | Across studies, serum copper varied widely: case means ranged from ~12 μmol/L to ~51 μmol/L (or ~101–478 μg/dL), controls from ~10 μmol/L to ~49 μmol/L, with a few extreme outliers. | Serum copper levels were significantly higher in cervical cancer patients compared to controls | ^75^ |
| Cervical Cancer | Fe (Ferritin serum concentration) | Prospective clinical study (n = 150; 98 untreated cervical cancer patients, 52 healthy female controls) | Japan | Cervical cancer cases (stages 0–IV, untreated) | Serum ferritin in 52 control women: mean 35 ng/mL; 2nd–98th percentile reference interval: 3.9–108 ng/mL. Upper limit of normal was defined as 108 ng/mL. | Elevated serum ferritin in cervical cancer patients was associated with parametrial invasion/metastasis and poor prognosis, suggesting utility in predicting disease extent and outcome. | ^28^ |
| Cervical Cancer | Se (plasma concentration) | Cross‑sectional case‑control study (n = 58; 29 poor prognosis cases with local recurrence or distant metastasis) | Harbin, China | Cervical cancer patients who had received treatment and were followed for recurrence or metastasis. | Plasma Se (mean ± SD): Poor prognosis 49.90 ± 13.81 µg/L; Good prognosis 59.38 ± 13.00 µg/L (t = 2.69, P = 0.009). | Plasma Se levels were significantly lower in poor prognosis patients compared to good prognosis patients | ^51^ |
| Cervical Cancer | Mn (serum and tissue concentrations) | Case-control study (serum: n = 300; 100 cervical cancer patients, 100 uterine myoma patients, 100 healthy controls; tissue: n = 70; 40 cervical cancer patients with paired tumor and non‑lesion tissue, 30 uterine myoma patients with paired myoma and non‑lesion tissue) | Taiyuan, Shanxi Province, China | Women aged 30–65 years (mean 45.7 years) with cervical cancer or uterine myoma undergoing surgery | Tissue Mn was lower in cervical cancer than in non‑lesion tissue (0.003±0.007 vs 0.006±0.011 μmol/g), with no myoma difference; serum Mn in cancer and myoma (both 0.003±0.003 mmol/L) was higher than in healthy controls | Tissue Mn was significantly lower in cervical cancer tissue compared to non‑lesion tissue. However, serum Mn was significantly higher in cervical cancer patients compared to healthy controls. | ^19^ |

**Supplementary Table 1 Clinical studies on trace element-cancer associations**

*Note.* Studies identified through structured searches of PubMed, Embase, and Web of Science, supplemented by reference list screening. Priority given to prospective/nested case-control designs with prediagnostic biospecimens, recent meta-analyses, and geographic diversity. Table is illustrative, not exhaustive.

**Abbreviations:** BMI, body mass index; BPH, benign prostatic hyperplasia; CI, confidence interval; CRC, colorectal cancer; Cu, copper; DFTJ, Dongfeng-Tongji cohort; DMC, Dongfeng Motor Corporation; EOC, epithelial ovarian cancer; EPIC, European Prospective Investigation into Cancer and Nutrition; ESCC, esophageal squamous cell carcinoma; Fe, iron; FFQ, food frequency questionnaire; FIGO, International Federation of Gynecology and Obstetrics; GLCC, Guangdong Liver Cancer Cohort; HCC, hepatocellular carcinoma; HR, hazard ratio; IQR, interquartile range; MCC-Spain, Multi-Case-Control Spain; mCRC, metastatic colorectal cancer; Mn, manganese; NAFLD, non-alcoholic fatty liver disease; NPC, Nutritional Prevention of Cancer; NSCLC, non-small cell lung cancer; OR, odds ratio; OS, overall survival; PCa, prostate cancer; PDAC, pancreatic ductal adenocarcinoma; PHS, Physicians' Health Study; PLCO, Prostate, Lung, Colorectal, and Ovarian; Q, quartile; RCT, randomized controlled trial; RR, relative risk; SCAN-B, Sweden Cancerome Analysis Network-Breast; SD, standard deviation; Se, selenium; SELECT, Selenium and Vitamin E Cancer Prevention Trial; SEP15, selenoprotein 15; SMD, standardized mean difference; T, tertile; WHI, Women's Health Initiative; ZD, zinc deficiency; Zn, zinc.

**References**

1	Abnet CC, Lai B, Qiao YL, Vogt S, Luo XM, Taylor PR *et al*. Zinc concentration in esophageal biopsy specimens measured by x-ray fluorescence and esophageal cancer risk. Journal of the National Cancer Institute 2005; 97: 301-306.

2	Aglago EK, Cross AJ, Riboli E, Fedirko V, Hughes DJ, Fournier A *et al*. Dietary intake of total, heme and non-heme iron and the risk of colorectal cancer in a European prospective cohort study. British journal of cancer 2023; 128: 1529-1540.

3	Allen NE, Appleby PN, Roddam AW, Tjønneland A, Johnsen NF, Overvad K *et al*. Plasma selenium concentration and prostate cancer risk: results from the European Prospective Investigation into Cancer and Nutrition (EPIC). The American journal of clinical nutrition 2008; 88: 1567-1575.

4	Amaral AF, Cantor KP, Silverman DT, Malats N. Selenium and bladder cancer risk: a meta-analysis. Cancer epidemiology, biomarkers & prevention : a publication of the American Association for Cancer Research, cosponsored by the American Society of Preventive Oncology 2010; 19: 2407-2415.

5	Augustyniak M, Galas A. Calcium intake may explain the reduction of colorectal cancer odds by dietary selenium - a case-control study in Poland. BMC nutrition 2022; 8: 22.

6	Bai Y, Cao Q, Guan X, Meng H, Feng Y, Wang C *et al*. Metabolic linkages between zinc exposure and lung cancer risk: A nested case-control study. The Science of the total environment 2022; 837: 155796.

7	Baker JR, Umesh S, Jenab M, Schomburg L, Tjønneland A, Olsen A *et al*. Prediagnostic Blood Selenium Status and Mortality among Patients with Colorectal Cancer in Western European Populations. Biomedicines 2021; 9.

8	Bao X, Yan L, Lin J, Chen Q, Chen L, Zhuang Z *et al*. Selenoprotein genetic variants may modify the association between serum selenium and oral cancer risk. Oral diseases 2020; 26: 1141-1148.

9	Bengtsson Y, Sandsveden M, Manjer J. Risk of breast cancer in relation to dietary intake of selenium and serum selenium as a marker of dietary intake: a prospective cohort study within The Malmö Diet and Cancer Study. Cancer causes & control : CCC 2021; 32: 815-826.

10	Bengtsson Y, Demircan K, Vallon-Christersson J, Malmberg M, Saal LH, Rydén L *et al*. Serum copper, zinc and copper/zinc ratio in relation to survival after breast cancer diagnosis: A prospective multicenter cohort study. Redox biology 2023; 63: 102728.

11	Byeon S, du Toit-Thompson T, Hipperson L, Maloney S, Wenzel R, Gill AJ *et al*. Serum and tissue metallome of pancreatic ductal adenocarcinoma. Cancer science 2024; 115: 1446-1458.

12	Caglayan A, Katlan DC, Tuncer ZS, Yüce K. Evaluation of trace elements associated with antioxidant enzymes in blood of primary epithelial ovarian cancer patients. Journal of trace elements in medicine and biology : organ of the Society for Minerals and Trace Elements (GMS) 2019; 52: 254-262.

13	Cai L, You NC, Lu H, Mu LN, Lu QY, Yu SZ *et al*. Dietary selenium intake, aldehyde dehydrogenase-2 and X-ray repair cross-complementing 1 genetic polymorphisms, and the risk of esophageal squamous cell carcinoma. Cancer 2006; 106: 2345-2354.

14	Cao B, Lei Y, Xue H, Liang Y, Liu Y, Xie Q *et al*. Changes in the Serum Concentrations of Essential Trace Metals in Patients with Benign and Malignant Breast Cancers. Biological trace element research 2022; 200: 3537-3544.

15	Chatterjee S, Combs GF, Jr., Chattopadhyay A, Stolzenberg-Solomon R. Serum selenium and pancreatic cancer: a prospective study in the Prostate, Lung, Colorectal and Ovarian Cancer Trial cohort. Cancer causes & control : CCC 2019; 30: 457-464.

16	Chen F, Wang J, Chen J, Yan L, Hu Z, Wu J *et al*. Serum copper and zinc levels and the risk of oral cancer: A new insight based on large-scale case-control study. Oral diseases 2019; 25: 80-86.

17	Chen XB, Wei YH, Chen XK, Zhong J, Zou YB, Nie JY. Manganese levels and hepatocellular carcinoma: A systematic review and meta-analysis based on Asian cohort. Medicine 2019; 98: e16748.

18	Cui Z, Liu D, Liu C, Liu G. Serum selenium levels and prostate cancer risk: A MOOSE-compliant meta-analysis. Medicine 2017; 96: e5944.

19	Cunzhi H, Jiexian J, Xianwen Z, Jingang G, Shumin Z, Lili D. Serum and tissue levels of six trace elements and copper/zinc ratio in patients with cervical cancer and uterine myoma. Biological trace element research 2003; 94: 113-122.

20	De Troy J, Fendt SM, Hatse S, Neven P, Smeets A, Laenen A *et al*. Plasma Iron Levels at Early Breast Cancer Diagnosis Are Associated With Development of Secondary Metastases: A Single-Center Retrospective Cohort Study. Breast cancer : basic and clinical research 2025; 19: 11782234251317070.

21	Demircan K, Bengtsson Y, Sun Q, Brange A, Vallon-Christersson J, Rijntjes E *et al*. Serum selenium, selenoprotein P and glutathione peroxidase 3 as predictors of mortality and recurrence following breast cancer diagnosis: A multicentre cohort study. Redox biology 2021; 47: 102145.

22	Devi V, Chaudhary V, Sharma M, Kumari S, Pal B. Serum levels of trace elements in patients with prostate cancer: a systematic review and meta-analysis. Cancer causes & control : CCC 2025; 36: 1307-1318.

23	Epstein MM, Kasperzyk JL, Andrén O, Giovannucci EL, Wolk A, Håkansson N *et al*. Dietary zinc and prostate cancer survival in a Swedish cohort. The American journal of clinical nutrition 2011; 93: 586-593.

24	Fritz H, Kennedy D, Fergusson D, Fernandes R, Cooley K, Seely A *et al*. Selenium and lung cancer: a systematic review and meta analysis. PloS one 2011; 6: e26259.

25	Gutiérrez-González E, Castelló A, Fernández-Navarro P, Castaño-Vinyals G, Llorca J, Salas D *et al*. Dietary Zinc and Risk of Prostate Cancer in Spain: MCC-Spain Study. Nutrients 2018; 11.

26	Hashemian M, Poustchi H, Abnet CC, Boffetta P, Dawsey SM, Brennan PJ *et al*. Dietary intake of minerals and risk of esophageal squamous cell carcinoma: results from the Golestan Cohort Study. The American journal of clinical nutrition 2015; 102: 102-108.

27	Hughes DJ, Duarte-Salles T, Hybsier S, Trichopoulou A, Stepien M, Aleksandrova K *et al*. Prediagnostic selenium status and hepatobiliary cancer risk in the European Prospective Investigation into Cancer and Nutrition cohort. The American journal of clinical nutrition 2016; 104: 406-414.

28	Ito H, Takagi Y, Ando Y, Kubo A, Hashimoto S, Tsutsui F *et al*. Serum ferritin levels in patients with cervical cancer. Obstetrics and gynecology 1980; 55: 358-362.

29	Jakszyn P, González CA, Luján-Barroso L, Ros MM, Bueno-de-Mesquita HB, Roswall N *et al*. Red meat, dietary nitrosamines, and heme iron and risk of bladder cancer in the European Prospective Investigation into Cancer and Nutrition (EPIC). Cancer epidemiology, biomarkers & prevention : a publication of the American Association for Cancer Research, cosponsored by the American Society of Preventive Oncology 2011; 20: 555-559.

30	Jin Y, Zhang C, Xu H, Xue S, Wang Y, Hou Y *et al*. Combined effects of serum trace metals and polymorphisms of CYP1A1 or GSTM1 on non-small cell lung cancer: a hospital based case-control study in China. Cancer epidemiology 2011; 35: 182-187.

31	Kasai F, Eshak ES, Tamakoshi A, Iso H. Dietary Manganese Intake and Risk of Liver Cancer in Japanese Men and Women: The JACC Study. Nutrition and cancer 2023; 75: 154-163.

32	Kluza M, Paszek S, Kluza K, Januszek S, Potocka N, Skrzypa M *et al*. An Assessment of Serum Selenium Concentration in Women with Ovarian Cancer. Nutrients 2023; 15.

33	Lee S, Song A, Eo W. Serum Ferritin as a Prognostic Biomarker for Survival in Relapsed or Refractory Metastatic Colorectal Cancer. Journal of Cancer 2016; 7: 957-964.

34	Lener MR, Scott RJ, Wiechowska-Kozłowska A, Serrano-Fernández P, Baszuk P, Jaworska-Bieniek K *et al*. Serum Concentrations of Selenium and Copper in Patients Diagnosed with Pancreatic Cancer. Cancer research and treatment 2016; 48: 1056-1064.

35	Li L, Gai X. The association between dietary zinc intake and risk of pancreatic cancer: a meta-analysis. Bioscience reports 2017; 37.

36	Lin S, Yang H. Ovarian cancer risk according to circulating zinc and copper concentrations: A meta-analysis and Mendelian randomization study. Clinical nutrition (Edinburgh, Scotland) 2021; 40: 2464-2468.

37	Liu F, Yang Q, Wang J, Wang Y, Weng Y, Lin Z *et al*. [Association between serum manganese level and oral cancer: a case-control study]. Wei sheng yan jiu = Journal of hygiene research 2025; 54: 79-86.

38	Liu X, Zhang Y, Yishake D, Luo Y, Liu Z, Chen Y *et al*. Dietary intake and serum levels of copper and zinc and risk of hepatocellular carcinoma: A matched case-control study. Chinese medical journal 2024; 137: 596-603.

39	M MM, Shetty P, Yajamanam N, Neralakatte SA, Hegde P, Shetty N *et al*. Evaluate the Role of Phosphodiesterase, Myeloperoxidase and Iron in Oral Cancer. Asian Pacific journal of cancer prevention : APJCP 2024; 25: 3921-3926.

40	Ma J, Li Q, Fang X, Chen L, Qiang Y, Wang J *et al*. Increased total iron and zinc intake and lower heme iron intake reduce the risk of esophageal cancer: A dose-response meta-analysis. Nutrition research (New York, NY) 2018; 59: 16-28.

41	Mahmoud AM, Al-Alem U, Dabbous F, Ali MM, Batai K, Shah E *et al*. Zinc Intake and Risk of Prostate Cancer: Case-Control Study and Meta-Analysis. PloS one 2016; 11: e0165956.

42	Mao S, Huang S. Zinc and copper levels in bladder cancer: a systematic review and meta-analysis. Biological trace element research 2013; 153: 5-10.

43	Mark SD, Qiao YL, Dawsey SM, Wu YP, Katki H, Gunter EW *et al*. Prospective study of serum selenium levels and incident esophageal and gastric cancers. Journal of the National Cancer Institute 2000; 92: 1753-1763.

44	Mazdak H, Yazdekhasti F, Movahedian A, Mirkheshti N, Shafieian M. The comparative study of serum iron, copper, and zinc levels between bladder cancer patients and a control group. International urology and nephrology 2010; 42: 89-93.

45	Milde D, Novák O, Stu ka V, Vyslou il K, Machá ek J. Serum levels of selenium, manganese, copper, and iron in colorectal cancer patients. Biological trace element research 2001; 79: 107-114.

46	Molina-Montes E, Wark PA, Sánchez MJ, Norat T, Jakszyn P, Luján-Barroso L *et al*. Dietary intake of iron, heme-iron and magnesium and pancreatic cancer risk in the European prospective investigation into cancer and nutrition cohort. International journal of cancer 2012; 131: E1134-1147.

47	Pal A, Dhar A, Shamim MA, Rani I, Negi RR, Sharma A *et al*. Selenium levels in colorectal cancer: A systematic review and meta-analysis of serum, plasma, and colorectal specimens. Journal of trace elements in medicine and biology : organ of the Society for Minerals and Trace Elements (GMS) 2024; 84: 127429.

48	Panaiyadiyan S, Quadri JA, Nayak B, Pandit S, Singh P, Sachan A *et al*. Association of heavy metals and trace elements in carcinoma urinary bladder: A case-controlled study. Indian journal of urology : IJU : journal of the Urological Society of India 2023; 39: 236-240.

49	Penney KL, Schumacher FR, Li H, Kraft P, Morris JS, Kurth T *et al*. A large prospective study of SEP15 genetic variation, interaction with plasma selenium levels, and prostate cancer risk and survival. Cancer prevention research (Philadelphia, Pa) 2010; 3: 604-610.

50	Pinto V, Marinaccio M, Garofalo S, Vittoria Larocca AM, Geusa S, Lanzilotti G *et al*. Preoperative evaluation of ferritinemia in primary epithelial ovarian cancer. Tumori 1997; 83: 927-929.

51	Qi L, Wang Y, Wang R, Wang M, Jablonska E, Zhou H *et al*. Association of Plasma Selenium and Its Untargeted Metabolomic Profiling with Cervical Cancer Prognosis. Biological trace element research 2023; 201: 4637-4648.

52	Qiu S, Xie B, Liao J, Luo J, Liu X, He L *et al*. Blood trace elements in association with esophageal squamous cell carcinoma risk, aggressiveness and prognosis in a high incidence region of China. Scientific reports 2025; 15: 5208.

53	Quintana Pacheco DA, Sookthai D, Graf ME, Schübel R, Johnson T, Katzke VA *et al*. Iron status in relation to cancer risk and mortality: Findings from a population-based prospective study. International journal of cancer 2018; 143: 561-569.

54	Rejali L, Jaafar MH, Ismail NH. Serum selenium level and other risk factors for breast cancer among patients in a Malaysian hospital. Environmental health and preventive medicine 2007; 12: 105-110.

55	Saleh SAK, Adly HM, Abdelkhaliq AA, Nassir AM. Serum Levels of Selenium, Zinc, Copper, Manganese, and Iron in Prostate Cancer Patients. Current urology 2020; 14: 44-49.

56	Seiki Y, Ikezawa K, Kai Y, Takada R, Kawabata M, Kishimoto H *et al*. Impact of zinc deficiency on the prognosis of unresectable pancreatic cancer. Pancreatology : official journal of the International Association of Pancreatology (IAP) [et al] 2025; 25: 552-557.

57	Shen F, Cai WS, Li JL, Feng Z, Cao J, Xu B. The association between deficient manganese levels and breast cancer: a meta-analysis. International journal of clinical and experimental medicine 2015; 8: 3671-3680.

58	Squitti R, Pal A, Dhar A, Shamim MA, Ventriglia M, Simonelli I *et al*. Serum copper status of patients with colorectal cancer: A systematic review and meta-analysis. Journal of trace elements in medicine and biology : organ of the Society for Minerals and Trace Elements (GMS) 2024; 82: 127370.

59	Sukiennicki GM, Marciniak W, Muszyńska M, Baszuk P, Gupta S, Białkowska K *et al*. Iron levels, genes involved in iron metabolism and antioxidative processes and lung cancer incidence. PloS one 2019; 14: e0208610.

60	Szwiec M, Marciniak W, Derkacz R, Huzarski T, Gronwald J, Cybulski C *et al*. Serum Selenium Level Predicts 10-Year Survival after Breast Cancer. Nutrients 2021; 13.

61	Szwiec M, Marciniak W, Derkacz R, Huzarski T, Gronwald J, Cybulski C *et al*. Serum Levels of Copper and Zinc and Survival in Breast Cancer Patients. Nutrients 2024; 16.

62	Takata Y, Kristal AR, King IB, Song X, Diamond AM, Foster CB *et al*. Serum selenium, genetic variation in selenoenzymes, and risk of colorectal cancer: primary analysis from the Women's Health Initiative Observational Study and meta-analysis. Cancer epidemiology, biomarkers & prevention : a publication of the American Association for Cancer Research, cosponsored by the American Society of Preventive Oncology 2011; 20: 1822-1830.

63	Tu K, Liu K, Wang Y, Jiang Y, Zhang C. Association of Dietary Intake of Zinc and Selenium with Breast Cancer Risk: A Case-Control Study in Chinese Women. Nutrients 2023; 15.

64	Wang Q, Cui Q, Gao JP, Xing R. Role of iron biomarkers and iron intakes in lung cancer risk: A systematic review and meta-analysis. Journal of trace elements in medicine and biology : organ of the Society for Minerals and Trace Elements (GMS) 2022; 74: 127060.

65	Wang W, Wang X, Luo J, Chen X, Ma K, He H *et al*. Serum Copper Level and the Copper-to-Zinc Ratio Could Be Useful in the Prediction of Lung Cancer and Its Prognosis: A Case-Control Study in Northeast China. Nutrition and cancer 2021; 73: 1908-1915.

66	Wang Y, Sun Z, Li A, Zhang Y. Association between serum zinc levels and lung cancer: a meta-analysis of observational studies. World journal of surgical oncology 2019; 17: 78.

67	Wei WQ, Abnet CC, Qiao YL, Dawsey SM, Dong ZW, Sun XD *et al*. Prospective study of serum selenium concentrations and esophageal and gastric cardia cancer, heart disease, stroke, and total death. The American journal of clinical nutrition 2004; 79: 80-85.

68	Wu X, Wu H, Liu L, Qiang G, Zhu J. Serum zinc level and tissue ZIP4 expression are related to the prognosis of patients with stages I-III colon cancer. Translational cancer research 2020; 9: 5585-5594.

69	Xie Y, Wang J, Zhao X, Zhou X, Nie X, Li C *et al*. Higher serum zinc levels may reduce the risk of cervical cancer in Asian women: A meta-analysis. The Journal of international medical research 2018; 46: 4898-4906.

70	Yu YC, Luu HN, Wang R, Thomas CE, Glynn NW, Youk AO *et al*. Serum Biomarkers of Iron Status and Risk of Hepatocellular Carcinoma Development in Patients with Nonalcoholic Fatty Liver Disease. Cancer epidemiology, biomarkers & prevention : a publication of the American Association for Cancer Research, cosponsored by the American Society of Preventive Oncology 2022; 31: 230-235.

71	Zachara BA, Marchaluk-Wiśniewska E, Maciag A, Pepliński J, Skokowski J, Lambrecht W. Decreased selenium concentration and glutathione peroxidase activity in blood and increase of these parameters in malignant tissue of lung cancer patients. Lung 1997; 175: 321-332.

72	Zhan M, Chuai Y, Zhou X, Li J, Zhou X, Zhang Z *et al*. A Case-Control Study of Association Between Serum Levels of 19 Trace Elements with Prostate Cancer in Southwest China. Biological trace element research 2025; 203: 5081-5095.

73	Zhang H, Xu Z, Zhang J, Wei D, Liu K, Hu W *et al*. Disordered serum essential element levels are associated with increased risk of kidney tumors. Environmental science and pollution research international 2022; 29: 31675-31685.

74	Zhang J, Wang G, Huang A, Cao K, Tan W, Geng H *et al*. Association between Serum Level of Multiple Trace Elements and Esophageal Squamous Cell Carcinoma Risk: A Case-Control Study in China. Cancers 2022; 14.

75	Zhang M, Shi M, Zhao Y. Association between serum copper levels and cervical cancer risk: a meta-analysis. Bioscience reports 2018; 38.

76	Złowocka-Perłowska E, Baszuk P, Marciniak W, Derkacz R, Tołoczko-Grabarek A, Gołębiewska K *et al*. Blood and Serum Copper and Zinc Levels and 10-Year Survival of Patients After Kidney Cancer Diagnosis. Nutrients 2025; 17.
